# A systematic review and meta-analysis of acute kidney injury in the intensive care units of developed and developing countries

**DOI:** 10.1371/journal.pone.0226325

**Published:** 2020-01-17

**Authors:** Fernando de Assis Ferreira Melo, Etienne Macedo, Ana Caroline Fonseca Bezerra, Walédya Araújo Lopes de Melo, Ravindra L. Mehta, Emmanuel de Almeida Burdmann, Dirce Maria Trevisan Zanetta

**Affiliations:** 1 Division of Urology, Acre Federal University, Rio Branco, Acre, Brazil; 2 Department of Medicine, University of California San Diego (UCSD), San Diego, California, United States of America; 3 LIM 12, Division of Nephrology, University of São Paulo Medical School, São Paulo, São Paulo, Brazil; 4 Department of Epidemiology, University of São Paulo School of Public Health, São Paulo, São Paulo, Brazil; Istituto Di Ricerche Farmacologiche Mario Negri, ITALY

## Abstract

**Objectives:**

Although the majority of the global population lives in developing countries, most of the epidemiological data related to intensive care unit (ICU) acute kidney injury (AKI) comes from developed countries. This systematic review aims to ascertain the methodology of studies on ICU AKI patients in developing and developed countries, to determine whether epidemiological comparisons between these two settings are possible, and to present a summary estimate of AKI incidence.

**Methods:**

A systematic review of published studies reporting AKI in intensive care units (2005–2015) identified in PubMed, LILACS, and IBECs databases was conducted. We compared developed and developing countries by evaluating study methodology, AKI reference serum creatinine definitions, population characteristics, AKI incidence and mortality. AKI incidence was calculated with a random-effects model.

**Results:**

Ninety-two studies were included, one of which reported data from both country categories: 60 from developed countries (1,057,332 patients) and 33 from developing countries (34,539 patients). In 78% of the studies, AKI was defined by the RIFLE, AKIN or KDIGO criteria. Oliguria had 11 different definitions and reference creatinine 23 different values. For the meta-analysis, 38 studies from developed and 18 from developing countries were selected, with similar AKI incidence: 39.3% and 35.1%, respectively. The need for dialysis, length of ICU stay and mortality were higher in developing countries.

**Conclusion:**

Although patient characteristics and AKI incidence were similar in developed and developing countries, main outcomes were worse in developing country studies. There are significant caveats when comparing AKI epidemiology in developed and developing countries, including lack of standardization of reference serum creatinine, oliguria and the timeframe for AKI assessment. Larger, prospective, multicenter studies from developing countries are urgently needed to capture AKI data from the overall population without ICU access.

## Introduction

Acute kidney injury (AKI) affects 20 to 50% of intensive care unit (ICU) patients, and it is associated with high mortality, increased ICU length of stay and greater hospitalization cost [1–5]. When renal replacement therapy (RRT) is used, mortality rates can reach up to 80% [2,6].

It is widely accepted that AKI characteristics are different in developed and developing countries due to contrasting socioeconomic patterns, government health expenditures, heath service infrastructure and AKI etiology [7].

Although the vast majority of the global population lives in developing countries, most of the epidemiological data from ICU patients with AKI comes from developed countries. Comparisons of these two different settings are scarce. A multicenter prospective study found higher mortality for ICU AKI patients in developing countries [8], which might be related to an inadequate number of ICU beds in relation to population size and the difficulty of health care access, among other reasons. To determine whether the outcomes in these populations are comparable, it is necessary to evaluate whether there are differences in patient characteristics as well as the methodological aspects among the analyzed studies.

This systematic review covers methodological aspects, including AKI and reference serum creatinine definitions, as well as the main characteristics and outcomes of ICU AKI patients from studies in developed and developing countries. We aim to determine whether epidemiological comparisons between these two country categories are appropriate with the available data and to estimate their AKI incidence using meta-analysis.

## Material and methods

### Database search

**Fig 1:**
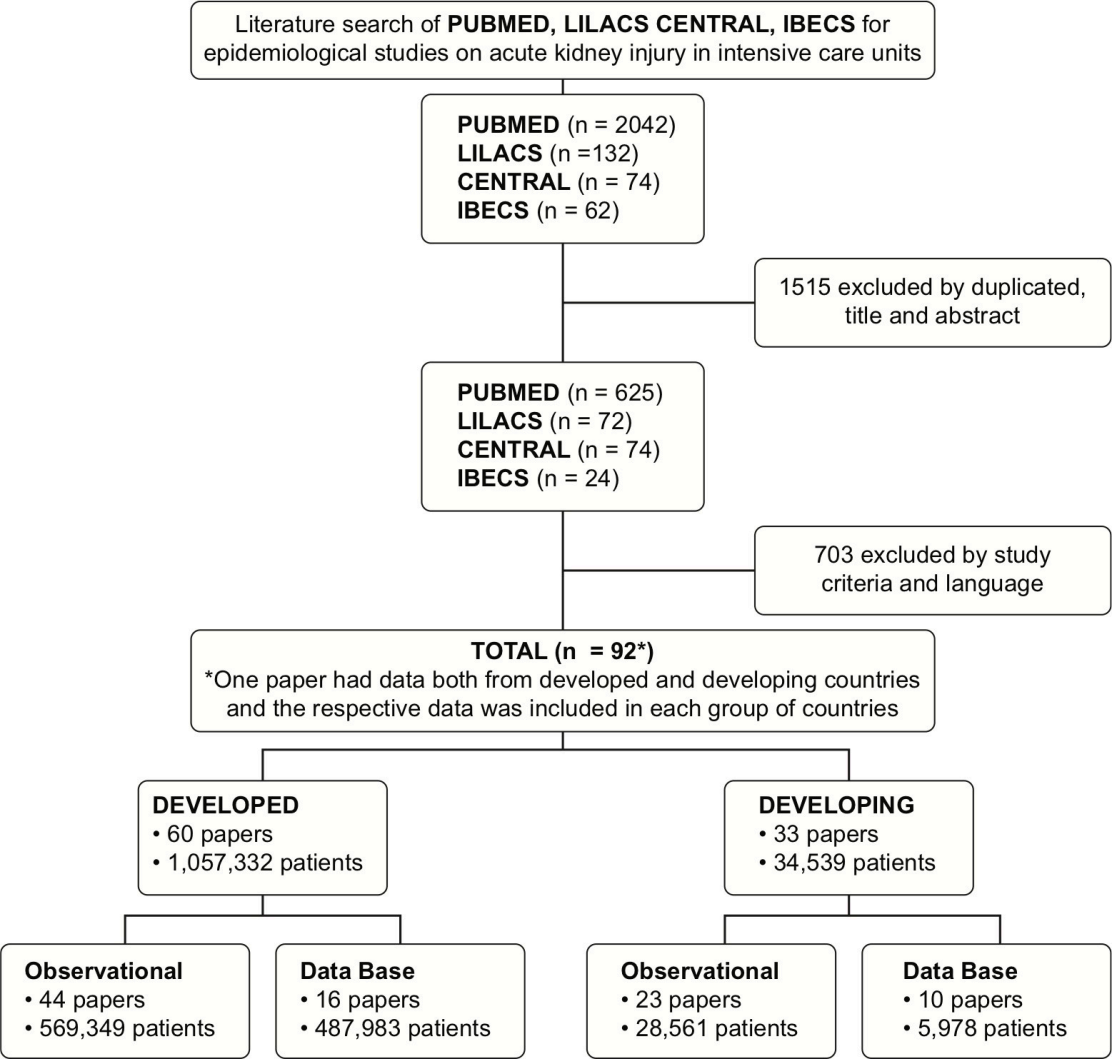
Flowchart of study selection.

This systematic review was conducted using the recommendations of the *“Cochrane Handbook for Systematic Reviews of Interventions”* [9] (Fig 1). A systematic electronic search was performed to identify all original studies which might include acute kidney injury patients in intensive care units published from 2005 until 2015, including the keyword terms: “acute kidney injury”, “acute kidney failure”, “acute kidney insufficiency”, “acute renal injury”, “acute renal failure”, “acute renal insufficiency”, “intensive care units”, “critical ill patient and critical ill”. We accessed the PubMed, CENTRAL (Cochrane Controlled Register of Trials), LILACS (Latin American and Caribbean Health Sciences Library), and IBECs (Spanish Bibliographical Index of Health Sciences) databases. The search strategies for each database can be found in S1 File. This search was last updated on July 31, 2015, and the language was limited to English, Spanish, French, Italian and Portuguese. The manuscripts were manually analyzed in order to find additional references. There was no blinding in relation to the author, place of publication, or journal.

The studies were classified into two groups: those from developed and those from developing countries, and they were assigned group membership based on the United Nations classification.

### Study selection

An initial eligibility screen of all retrieved titles and abstracts was conducted, and only studies reporting AKI in intensive care units in the 10-year surveyed period were selected for further review. Full-text papers were obtained for inspection of each study that potentially fulfilled the inclusion criteria on the basis of title and/or abstract. The following specific criteria were used for final selection: only studies with adult patients and those with reported epidemiological data. Duplicate articles were identified and eliminated using the Endnoteweb [10] software tool.

The final group of manuscripts was selected by the main author and reviewed by a coauthor. Discordances were solved by consensus using the predefined inclusion and exclusion criteria in accordance with the recommendations of the *“Cochrane Handbook for Systematic Reviews of Interventions*” [9]. When necessary, a final decision was achieved by consulting a third coauthor.

### Data collection process

Two authors performed independent data extraction using standardized data extraction forms.

The following data were retrieved:

1. **Research place and study description**: country, study type, length of data collection, number of ICUs, type of ICU, number of patients.
2. **AKI characteristics**: incidence, definition, criteria used for definition, timeframe for AKI assessment, percentage of oliguric patients, oliguria definition, reference serum creatinine (SCr) defined as the value used for comparing increased SCr to establish the diagnosis of AKI.
3. **AKI patient characteristics**: mean age, gender ratio, previous comorbidities, patient source, severity of illness score.
4. **Outcomes:** length of ICU stay, length of hospital stay, frequency of RRT use, type of RRT, mortality.

### Statistical analysis

The frequencies of studies were calculated by considering those that assessed the respective data. The number of studies used for each calculation is shown after the presented frequency.

Weighted means and percentages of reported data were estimated using the study population as weight.

The pooled point AKI incidence of developed and developing countries´ studies was estimated for studies that used the RIFLE, AKIN or KDIGO criteria for the AKI definition. All estimates and their 95% confidence intervals (95% CI) were calculated using a random-effects model for descriptive data analysis. Subgroup analyses were conducted with studies grouped by each different criterion for the utilized AKI definition. Heterogeneity and consistency were evaluated using Cochran’s Q and the I^2^ statistics, respectively. Funnel plots were used to evaluate publication bias. The analysis was performed in Microsoft Excel, using the step-by-step approach constructed by Neyeloff et al. [11] to analyze descriptive data. The pooled AKI incidence of both country groups were compared using the estimated 95% confidence interval.

## Results

Of 2,459 potential studies, 1,635 were excluded because they did not assess patients, were duplicates, or had titles and abstracts that were not related to the purpose of this review. Of the remaining 824 studies, 17 were excluded for not meeting the language definitions and 714 were excluded as they did not meet the eligibility criteria. Ninety-two studies were included in the final analysis. One study [8] reported data from both developed and developing countries, and its data was reported in both country categories. As a result, the final analysis consisted of 60 studies presenting data from developed countries and 33 studies with data from developing countries (Fig 1 and Table 1). The 92 studies included in the final analysis can be found in S2 File.

**Table 1:** Frequency of studies in developed and developing countries with data reported.

	Developed countries	Developing countries
	(N = 60)	(N = 33)
**Description of studies (n = 93)**		
Retrospective	46.7% (28)	42.4% (14)
Prospective	53.3% (32)	57.6% (19)
**Definition of AKI by**		
RIFLE	34.4% (20)	39.3% (13)
AKIN	37.9% (22)	33.3% (11)
KDIGO	10% (6)	21.2% (7)
sCr* rise	24.1% (14)	9% (3)
sCr rise + decreases in urinary volume	73.2% (41)	78.1% 25
**Oliguria definition (n = 81)**	81.6% (49)	97% (32)
**Reference SCr definition (n = 60)**	68.3% (41)	57.5% (19)
**Timeframe for AKI diagnosis**	58.3% (35)	69.6% (23)
**Frequency of AKI > 40%**	54.7% (29)	82.1% (23)
**AKI etiology described**	46.6% (28)	66.6% (22)
**Patient characteristics**		
> 65 years old	40% (22)	12.1% (4)
Male frequency > 60%	59.6% (34)	67.8% (19)
Comorbidities	51.6% (31)	78.7% (26)
Severity scores		
APACHE II	58.3% (35)	54.5% (18)
SOFA	25% (15)	18.1% (6)
**Outcomes**		
Length of ICU stay	71.6% (43)	69.6% (23)
Length of hospital stay	38.3% (23)	21.2% (7)
RRT > 30% in AKI patients	13.3% (8)	30.3% (10)
Mortality	15.9% (7)	56% (14)

*sCr = serum creatinine

### Description of studies

#### Study design

In both developed and developing countries, the majority of studies were from university hospitals [91.6% (55/60) and 72.7% (24/33), respectively] (Tables 2 and 3).

**Table 2:** Country and study features description—developed countries.

Author	Home country	Study type	Length of data collection	Number of ICUs	Type of ICU	Number of Patients
Abosaif et al, 2005	England	Retrospective	24	1	Mixed	247
Bagshaw et al, 2005	Canada	Prospective	36	3	Mixed	5,693
Chawla et al, 2005	USA	Prospective	8	1	Mixed	194
Ostermann et al, 2005	England and Germany	Retrospective	110	22	Mixed	41,972
Ahlstrom et al, 2006	Finland	Prospective	11	2	Mixed	658
Herrera-Gutiérrez et al, 2006	Spain	Prospective	8	43	Mixed	15,714
Hoste, 2006	USA	Retrospective	12	7	Mixed	5,383
Bagshaw et al, 2007	Australia	Prospective	120	20	Mixed	91,254
Cruz et al, 2007	Italy	Prospective	3	19	Mixed	2,164
Eachempati et al, 2007	USA	Prospective	108	1	Mixed	41,972
Ostermann et al, 2007	England and Germany	Retrospective	120	11	Surgery	8,505
Bagshaw et al, 2008	Australia	Retrospective	60	57	Trauma	124,088
Bagshaw et al, 2008	Australia	Retrospective	60	57	Mixed	120,123
Barrantes et al, 2008	USA	Retrospective	12	1	Mixed	471
Lopes et al, 2008	Portugal	Retrospective	36	1	Mixed	662
Ostermann et al, 2008	England and Germany	Retrospective	123	22	Mixed	23,303
Abelha et al, 2009	Portugal	Retrospective	24	1	Mixed	1,166
Andrikos et al, 2009	Italy and Greece	Prospective	4	22	Mixed	1,076
Cartin-Ceba et al, 2009	USA	Retrospective	42	3	Mixed	11,644
Costantini et al, 2009	USA	Retrospective	36	1	Surgery	571
Joannidis et al, 2009	Austria and Portugal	Prospective	. . .	303	Mixed	16,784
Thakar et al, 2009	USA	Retrospective	60	191	Mixed	325,395
Aldawood et al, 2010	Saudi Arabia	Retrospective	72	1	Mixed	7,173
Cruz et al, 2010	USA	Prospective	6	1	Mixed	301
Elseviers et al, 2010	Belgium	Prospective	35	9	Mixed	1,303
Park et al, 2010	Korea	Retrospective	6	1	Mixed	378
Clec'h et al, 2011	France	Retrospective	149	13	Mixed	8,639
Darmon et al, 2011	France	Prospective	5	3	Mixed	203
Garzotto et al, 2011	Italy	Prospective	7	10	Mixed	576
Macedo et al, 2011	USA	Prospective	2	1	Mixed	75
Macedo et al, 2011	USA	Prospective	. . .	1	Mixed	317
Mandelbaum et al, 2011	USA	Retrospective	72	7	Mixed	14,524
Medve et al, 2011	Hungary	Prospective	2	7	Mixed	459
Ostermann et al, 2011	England and Germany	Retrospective	123	22	Mixed	22,303
Piccini et al, 2011	Italy	Prospective	7	10	Mixed	576
Prowle et al, 2011	Australia, Canada, Japan, USA, Germany, Italy	Prospective	1	7	Mixed	239
Clark et al, 2012	Canada	Prospective	11	11	Mixed	119
Han et al, 2012	Korea	Retrospective	57	1	Mixed	1625
Medve et al, 2012	Hungary	Prospective	. . .	1	Surgery	265
Odutayo et al, 2012	Canada	Prospective	12	5	Mixed	603
Shashaty et al, 2012	USA	Retrospective	45	1	Trauma	400
Sigurdsson et al, 2012	Iceland	Retrospective	12	2	Mixed	1012
Vaara et al, 2012	Finland	Retrospective	23	9	Mixed	30,380
Wohlauer et al, 2012	USA	Prospective	192	1	Surgery	2,158
Allegretti et al, 2013	USA	Retrospective	44	1	Mixed	863
Alsultan et al, 2013	Saudi Arabia	Prospective	36	2	Mixed	2,574
Fuchs et al, 2013	USA	Retrospective	80	1	Mixed	12,339
Legrand et al, 2013	France	Retrospective	48	1	Surgery	137
Nisula et al, 2013	Finland	Prospective	6	10	Mixed	1568
Poukkanen et al, 2013	Finland	Prospective	5	1	Infectious diseases	423
Poukkanen et al, 2013	Finland	Prospective	5	1	Infectious diseases	918
Doi et al, 2014	Japan	Prospective	5	1	Mixed	339
Han et al, 2014	Korea	Retrospective	72	1	Mixed	1,883
Linder et al, 2014	Canada	Prospective	108	1	Mixed	1,844
Shinjo et al, 2014	Japan	Retrospective	60	1	Mixed	2,579
Udy et al, 2014	Australia, Singapore, Hong Kong and Portugal	Prospective	. . .	4	Mixed	281
Bouchard et al, 2015	Various	Prospective	20	9	Mixed	316
Harris et al, 2015	USA	Retrospective	12	1	Surgery	624
Rimes-Stigiare et al, 2015	Sweden	Prospective	72	41	Mixed	97,782
Vanmassenhove et al, 2015	Belgium	Prospective	14	1	Mixed	195

**Table 3:** Country and study features description—developing countries.

Author	Home country	Study type	Length of data collection	Number of ICUs	Type of ICU	Number of patients
Mataloun et al, 2006	Brazil	Prospective	12	1	Mixed	221
Silva Junior et al, 2006	Brazil	Retrospective	48	1	Mixed	381
Chow et al, 2007	Malaysia	Prospective	6	1	Mixed	18,697
Daher et al, 2008	Brazil	Retrospective	36	1	Infectious diseases	722
Lima et al, 2008	Brazil	Retrospective	36	1	Infectious diseases	829
Fernandes et al, 2009	Brazil	Prospective	10	1	Mixed	89
Friedericksen et al, 2009	South Africa	Retrospective	12	1	Mixed	198
Chang et al, 2010	Taiwan	Retrospective	35	1	Mixed	291
Maccariello et al, 2010	Brazil	Prospective	18	11	Mixed	244
Balushi et al, 2011	Oman	Retrospective	12	1	Mixed	1,373
Ponce et al, 2011	Brazil	Prospective	24	1	Mixed	564
Fonseca Ruiz et al, 2011	Colombia	Retrospective	24	1	Mixed	794
Samimagham et al, 2011	Iran	Retrospective	12	1	Mixed	235
Alves et al, 2012	Brazil	Retrospective	15	1	Mixed	204
Chen et al, 2012	Taiwan	Prospective	12	1	Mixed	150
Daher et al, 2012	Brazil	Prospective	12	1	Mixed	408
Lai et al, 2012	Taiwan	Retrospective	101	1	Surgical	634
Wahrhaftig et al, 2012	Brazil	Prospective	12	1	Mixed	200
Zhou et al, 2012	China	Retrospective	8	5	Mixed	1,036
Dalboni et al, 2013	Brazil	Prospective	. . .	1	Mixed	303
Levi et al, 2013	Brazil	Prospective	12	1	Mixed	190
Silva et al, 2013	Brazil	Prospective	20	6	Mixed	366
Singh et al, 2013	India	Prospective	17	1	Mixed	1,504
Daher et al, 2014	Brazil	Retrospective	99	1	Infectious diseases	253
Luo et al, 2014	China	Prospective	6	30	Mixed	3,107
Morales-Buenrostro et al, 2014	Mexico	Prospective	3	1	Mixed	56
Peng et al, 2014	China	Retrospective	36	1	Infectious diseases	211
Wijewickrama et al, 2014	Sri Lanka	Prospective	6	1	Mixed	108
Bentata et al, 2015	Morocco	Retrospective	84	1	Obstetric	186
Bouchard et al, 2015	Various	Prospective	20	5	Mixed	429
Heegard et al, 2015	Afghanistan	Prospective	18	2	Trauma	134
Ralib et al, 2015	Malaysia	Prospective	3	1	Mixed	143
Santos et al, 2015	Brazil	Prospective	12	1	Mixed	279

The 92 studies report data from 1,091,871 patients. The number of patients from developed countries was 30 times higher; 1,057,332 vs. 34,539 patients in developed versus developing countries, respectively. Larger cohorts with more than 5,000 patients were more frequent in developed countries [33.3% (20/60)], whereas in developing countries, 87.8% (29/33) of the studies included less than 1,000 patients (Tables 2 and 3). The number of ICUs included in developed countries was significantly higher than in developing countries (990 vs. 86 ICUs). In fact, in developed countries, 41.6% of the studies (25/60) assessed more than five ICUs, while in developing countries, 81.8% of studies (27/33) assessed only one ICU. The majority of studies (82.7%, i.e., 77/93) evaluated patients from ICUs classified as "mixed" (Tables 2 and 3).

#### Definition of AKI

**Fig 2:**
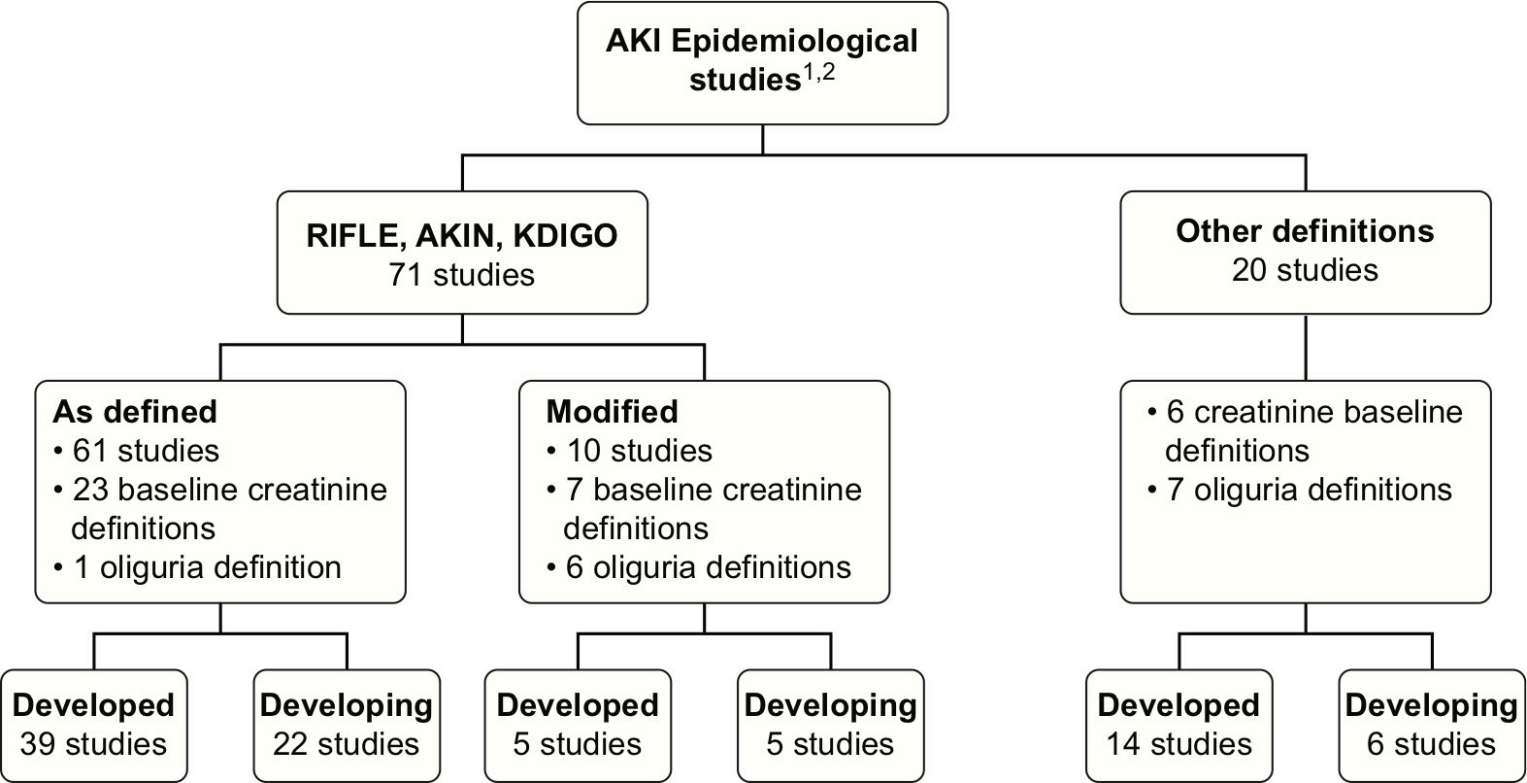
Flowchart of AKI definition criteria. Footnote: 1 One study contained data from both (developed and developing) country groups, and the respective data were included in each country group for the analysis. 2 Two studies did not mention the criteria used for the definition of IRA.

Both developed and developing country studies frequently used RIFLE, AKIN, KDIGO, as defined or modified (Fig 2). In developed countries, AKIN and RIFLE were the most frequently used criteria [37.9% (22/58) and 34.4%, (20/58), respectively], followed by increased SCr (24.1%, 14/58). In developing countries, RIFLE and AKIN were also more often applied [39.3% (13/33) and 33.3% (11/33), respectively], followed by KDIGO at 21.2% (7/33) (Tables 4 and 5). The majority of studies [73.2% (41/56) in developed countries and 78.1% (25/32) in developing countries] reported using both increases in SCr and decreases in urinary volume for the AKI diagnosis.

**Table 4:** AKI characteristics—developed countries.

Author	AKI Frequency (%)	AKI definition	Criteria used for AKI definition	Timeframe for AKI assessment	Oliguric patients (%)	Oliguria definition	Reference creatinine definition
Abosaif et al, 2005	. . .	RIFLE	Cr	. . .	. . .	< 0.5 ml/kg/h x 6 h	Lower Cr on hospital admission
Bagshaw et al, 2005	4.2	Cr rise/Oliguria	Cr/Diuresis	. . .	77.0	< 500 ml x 24 h	. . .
Chawla et al, 2005	18.0	Cr rise/Oliguria	Cr/Diuresis	Since ICU discharge or death	. . .	< 0.5 ml/kg/h x 48 h	. . .
Ostermann et al, 2005	17.9	Cr rise/Oliguria	Cr/Diuresis	. . .	. . .	BUN > 8 mmol/l	Cr > 120 mmol/l
Ahlstrom et al, 2006	51.9	RIFLE	Cr	Since ICU discharge or death	. . .	< 0.5 ml/kg/h x 6 h	MDRD
Herrera-Gutiérrez et al, 2006	5.7	Cr rise/Oliguria	Cr/Diuresis	. . .	. . .	< 400 ml x 24 h	. . .
Hoste et al, 2006	67.0	RIFLE	Cr/Diuresis	. . .	. . .	< 0.5 ml/kg/h x 6 h	Lower Cr on hospital admission, or MDRD
Bagshaw et al, 2007	5.2	Cr rise/Oliguria	Cr/Diuresis	. . .	. . .	. . .	. . .
Cruz et al, 2007	10.8	RIFLE	Cr/Diuresis	Since ICU discharge or death	. . .	< 0.5 ml/kg/h x 6 h	MDRD
Eachempati et al, 2007	6.2	Cr rise	Cr		. . .	. . .	Cr > 2.4 mg/dl
Ostermann et al, 2007	35.8	RIFLE	Cr	Since ICU discharge or death	. . .	< 0.5 ml/kg/h x 6 h	. . .
Bagshaw et al, 2008	36.1	RIFLE modified	Cr/Diuresis	Since ICU discharge or death, after 24 h from ICU admission	. . .	< 35 ml/kg/h	MDRD
Bagshaw et al, 2008	36.1/37.1	RIFLE modified/AKIN modified	Cr/Diuresis	Since ICU discharge or death, after 24 h from ICU admission	. . .	< 35 ml/kg/h	MDRD
Barrantes et al, 2008	42.9	AKIN	Cr/Diuresis	. . .	. . .	< 0.5 ml/kg/h x 6 h	. . .
Lopes et al, 2008	43.8/50.4	RIFLE modified/AKIN modified	Cr/Diuresis	Until 48 h	. . .	< 7.2 ml/kg X 24 h	MDRD
Ostermann et al, 2008	35.4	Cr rise	Cr	. . .	. . .	. . .	. . .
Abelha et al, 2009	7.5	AKIN	Cr/Diuresis	. . .	. . .	< 0.5 ml/kg/h x 6 h	. . .
Andrikos et al, 2009	16.0	RIFLE	Cr/Diuresis	. . .	. . .	< 0.5 ml/kg/h x 6 h	. . .
Cartin-Ceba et al, 2009	50.0	RIFLE	Cr/Diuresis	Since ICU discharge or death	. . .	< 0.5 ml/kg/h x 6 h	Lower Cr 3 months before ICU admission
Costantini et al, 2009	29.8	AKIN	Cr/Diuresis	Since ICU discharge or death after 48 h from admission	. . .	< 0.5 ml/kg/h x 6 h	. . .
Joannidis et al, 2009	35.5/28.5	RIFLE/AKIN	Cr/Diuresis	7 days	. . .	< 0.5 ml/kg/h x 6 h	MDRD
Thakar et al, 2009	60.8	AKIN modified	Cr	Since ICU discharge or death	. . .	. . .	Lower Cr 24 h before hospital admission
Aldawood et al, 2010	24.4	. . .	. . .	Since hospital discharge or death	. . .	. . .	. . .
Cruz et al, 2010	44.0	Cr rise	Cr/Diuresis	. . .	. . .	< 0.5 ml/kg/h x 6 h	Lower Cr 3 months before ICU admission or MDRD
Elseviers et al, 2010	. . .	Cr rise	Cr	. . .	. . .	. . .	. . .
Park et al, 2010	41.3	RIFLE	Cr/Diuresis	7 days	. . .	< 0.5 ml/kg/h x 6 h	Lower Cr 3 months before hospital admission, or MDRD
Clec’h et al, 2011	32.9	RIFLE	Cr/Diuresis	Since ICU discharge or death	. . .	< 0.5 ml/kg/h x 6 h	MDRD
Darmon et al, 2011	67.0	AKIN	Cr/Diuresis	12 hours	. . .	< 0.5 ml/kg/h x 6 h	. . .
Garzotto et al, 2011	42.7	RIFLE	Cr/Diuresis	. . .	. . .	< 0.5 ml/kg/h x 6 h	Lower Cr 3 months before ICU admission
Macedo et al, 2011	60.0	AKIN	Cr/Diuresis	Since ICU discharge or death	55.0	< 0.5 ml/kg/h x 6 h	First Cr on ICU admission
Macedo et al, 2011	52.0	AKIN	Cr/Diuresis	Since hospital discharge or death	47.0	< 0.5 ml/kg/h x 6 h	First Cr on ICU admission
Mandelbaum et al, 2011	57.0	AKIN	Cr/Diuresis	Since ICU discharge or death	. . .	< 0.5 ml/kg/h x 6 h	. . .
Medve et al, 2011	24.4	AKIN	Cr/Diuresis	Since ICU discharge or death	. . .	< 0.5 ml/kg/h x 6 h	First Cr on ICU admission
Ostermann et al, 2011	35.4	AKIN	Cr/Diuresis	Since ICU discharge or death	. . .	. . .	First Cr on ICU admission
Piccini et al, 2011	42.7	RIFLE	Cr/Diuresis	Since ICU discharge or death	. . .	< 0.5 ml/kg/h x 6 h	Lowest Cr 3 months before ICU admission
Prowle et al, 2011	9.6	AKIN modified/ RIFLE modified	Cr/Diuresis	4 weeks	38.0	< 0.5 ml/kg	Cr before disease or estimated using GRF 75 ml/min
Clark et al, 2012	7.8	AKIN	Cr	. . .	. . .	< 0.5 ml/kg/h x 6 h	Newest Cr 6 months before ICU admission, or MDRD
Han et al, 2012	57.0	AKIN	Cr/Diuresis	7 days	. . .	< 0.5 ml/kg/h x 6 h	Lowest Cr 7 days before hospital admission
Medve et al, 2012	18.1	AKIN	Cr/Diuresis	. . .	. . .	< 0.5 ml/kg/h x 6 h	First Cr on ICU admission
Odutayo et al, 2012	26.7	AKIN	Cr	90 days	. . .	< 0.5 ml/kg/h x 6 h	First Cr on ICU admission
Shashaty et al, 2012	36.8	AKIN	Cr/Diuresis	5 days	. . .	< 0.5 ml/kg/h x 6 h	First Cr on ICU admission
Sigurdsson et al, 2012	21.7	RIFLE	. . .	. . .	. . .	< 0.5 ml/kg/h x 6 h	Cr 1 year before ICU admission or the lowest after hospital discharge or MDRD
Vaara et al, 2012	26.6	RIFLE	Cr	Since ICU discharge or death	. . .	< 0.5 ml/kg/h x 6 h	Lowest Cr during ICU stay
Wohlauer et al, 2012	2.1	Cr rise	Cr	Since hospital discharge or death	. . .	. . .	. . .
Allegretti et al, 2013	. . .	Cr rise	Cr	. . .	. . .	. . .	First Cr on ICU admission
Alsultan et al, 2013	65.0	. . .	. . .	. . .	. . .	. . .	. . .
Fuchs et al, 2013	54.3	AKIN	Cr/Diuresis	. . .	. . .	< 0.5 ml/kg/h x 6 h	Lowest Cr on hospital admission
Legrand et al, 2013	50.3	AKIN	Cr/Diuresis	5 days	. . .	< 0.5 ml/kg/h x 6 h	Lowest Cr before ICU admission (time not informed), or MDRD
Nisula et al, 2013	40,5	KDIGO	Cr/Diuresis	5 days after 24 h from admission	. . .	< 0.5 ml/kg/h x 6 h	Newest Cr from last year, excluding the last week before ICU admission, or MDRD
Poukkanen et al, 2013	36.2	KDIGO	Cr/Diuresis	5 days	. . .	< 0.5 ml/kg/h x 6 h	Newest Cr from last year, excluding the week before ICU admission
Poukkanen et al, 2013	53.2	KDIGO	Cr/Diuresis	5 days	. . .	< 0.5 ml/kg/h x 6 h	Lowest Cr 1 year before ICU admission
Doi et al, 2014	38.6	RIFLE	Cr	. . .	. . .	< 0.5 ml/kg/h x 6 h	Lowest Cr 3 months before ICU admission or MDRD
Han et al, 2014	78.7	KDIGO	Cr/Diuresis	. . .	31.8	< 0.5 ml/kg/h x 6 h	. . .
Linder et al, 2014	57.0	KDIGO	Cr/Diuresis	Since ICU discharge or death	. . .	< 0.5 ml/kg/h x 6 h	Lowest Cr 3 months before ICU admission
Shinjo et al, 2014	29.5/38.4	AKIN/KDIGO	Cr/Diuresis	. . .	. . .	< 0.5 ml/kg/h x 6 h	Lowest Cr 3 months before ICU admission
Udy et al, 2014	65.1	Cr rise	Cr/Diuresis	Since ICU discharge or death, after 48 h from admission	. . .	. . .	. . .
Bouchard et al, 2015	19.1	AKIN	Cr/Diuresis	7 days	31.5	< 0.5 ml/kg/h x 6 h	Cr from 3 to 12 months before ICU admission
Harris et al, 2015	47.0	RIFLE	Cr	. . .	. . .	< 0.5 ml/kg/h x 6 h	Lowest Cr 1 year before ICU admission or MDRD
Rimes-Stigiare et al, 2015	5.4	Cr/Oliguria	Cr/Diuresis	. . .	. . .	< 400 ml x 24 h	. . .
Vanmassenhove et al, 2015	63.0	Cr rise	Cr	. . .	. . .	. . .	MDRD

* Cr = serum creatinine

**Table 5:** AKI characteristics—developing countries.

Author	AKI Frequency (%)	AKI definition	Criteria used for AKI definition	Timeframe for AKI assessment	Oliguric patients (%)	Oliguria definition	Reference creatinine definition
Mataloun et al, 2006	19.0	Cr rise	Cr	Since ICU discharge or death	. . .	. . .	. . .
Silva Junior et al, 2006	33.5	Cr rise	Cr	. . .	. . .	< 600 ml x 24 h	. . .
Chow et al, 2007	1.1	Oliguria	Diuresis	. . .	. . .	< 400 ml x 24 h	
Daher et al, 2008	20.3	RIFLE modified	Cr/Diuresis	Since ICU discharge or death	34.0	< 400 ml x 24 h	Lowest Cr on hospital admission, or MDRD
Lima et al, 2008	17.7	RIFLE	Cr/Diuresis	Since ICU discharge or death	. . .	< 0.5 ml/kg/h x 6 h	. . .
Fernandes et al, 2009	. . .	ATN-ISS	Diuresis	Since ICU discharge or death	62.0	< 400 ml x 24 h	Lowest Cr on hospital admission
Friedericksen et al, 2009	23.2	Cr rise	Cr	. . .	50.0	< 400 ml x 24 h	Lowest Cr on hospital admission
Chang et al, 2010	. . .	RIFLE/AKIN	Cr/Diuresis	. . .	. . .	< 0.5 ml/kg/h x 6 h	. . .
Maccariello et al, 2010	39.8	RIFLE	Cr/Diuresis	. . .	. . .	< 0.5 ml/kg/h x 6 h	. . .
Balushi et al, 2011	0.5	Cr / Oliguria	Cr/Diuresis	. . .	. . .	< 500 ml x 24 h	. . .
Ponce et al, 2011	25.5	AKIN	Cr/Diuresis	Since ICU discharge or death	68.5	< 0.5 ml/kg/h x 6 h	. . .
Fonseca Ruiz et al, 2011	31.1	AKIN	Cr/Diuresis	Since ICU discharge or death	. . .	< 0.5 ml/kg/h x 6 h	. . .
Samimagham et al, 2011	31.1	AKIN	Cr/Diuresis	Since ICU discharge or death	. . .	< 0.5 ml/kg/h x 6 h	. . .
Alves et al, 2012	11.8	RIFLE modified/AKIN modified	Cr/Diuresis	Since ICU discharge or death	. . .	< 400 ml x 6 h	Last Cr 6 months before ICU admission or MDRD
Chen et al, 2012	28.7	AKIN	Cr/Diuresis	. . .	. . .	< 0.5 ml/kg/h x 6 h	Cr on hospital admission
Daher et al, 2012	15.3	RIFLE modified	Cr/Diuresis	Since ICU discharge or death, after 24 h from admission	. . .	< 400 ml x 24 h	Lowest Cr before hospital admission or MDRD
Lai et al, 2012	. . .	RIFLE	Cr/Diuresis	Since ICU discharge or death	. . .	< 0.5 ml/kg/h x 6 h	Last Cr within 1 month to 1 year before admission
Wahrhaftig et al, 2012	36.0	RIFLE modified	Cr/Diuresis	Since ICU discharge or death	. . .	< 0.5 ml/kg/h x 6 h	Lowest Cr before hospital admission or MDRD
Zhou et al, 2012	34.1	AKIN	Cr/Diuresis	. . .	. . .	< 0.5 ml/kg/h x 6 h	Lowest Cr 2 days before ICU admission
Dalboni et al, 2013	45.6	RIFLE	Cr	48 h	. . .	< 0.5 ml/kg/h x 6 h	First Cr on ICU admission
Levi et al, 2013	. . .	KDIGO	Cr/Diuresis	Since ICU discharge or death, after 24 h from admission	. . .	< 0.5 ml/kg/h x 6 h	. . .
Silva et al, 2013	13.3	RIFLE	Cr	Since ICU discharge or death, after 48 h from admission	. . .	< 0.5 ml/kg/h x 6 h	. . .
Singh et al, 2013	2.2	RIFLE modified	Cr/Diuresis	Since hospital discharge or death, after 48 h from admission	61.0	< 400 ml x 24 h	Lowest Cr on hospital admission
Daher et al, 2014	. . .	RIFLE modified	Cr/Diuresis	Since ICU discharge or death	. . .	< 400 ml x 24 h	Lowest Cr before hospital admission or MDRD
Luo et al, 2014	46.9/38.4/51	RIFLE/AKIN/KDIGO	Cr/Diuresis	10 days	. . .	< 0.5 ml/kg/h x 6 h	Lower Cr 3 month before ICU admission
Morales-Buenrostro et al, 2014	30.3	AKIN		30 days	. . .	< 0.5 ml/kg/h x 6 h	First Cr on ICU admission
Peng et al, 2014	47.9	KDIGO	Cr/Diuresis	. . .	. . .	< 0.5 ml/kg/h x 6 h	. . .
Wijewickrama et al, 2014	60.2	AKIN	Cr/Diuresis	. . .	. . .	< 0.5 ml/kg/h x 6 h	. . .
Bentata et al, 2015	34.4	KDIGO	Cr/Diuresis	30 days	17.4	< 0.5 ml/kg/h x 6 h	First Cr on ICU admission
Bouchard et al, 2015	19.9	AKIN	Cr/Diuresis	7 days	26.1	< 0.5 ml/kg/h x 6 h	Cr from 3 to 12 months before hospital admission
Heegard et al, 2015	34.3	KDIGO	Cr/Diuresis	14 days	. . .	< 0.5 ml/kg/h x 6 h	Cr 1 year before hospital admission or MDRD
Ralib et al, 2015	65.0	KDIGO	Cr/Diuresis	Since ICU discharge or death, after 48 h from admission	61.0	< 0.5 ml/kg/h x 6 h	First Cr on ICU admission, or MDRD
Santos et al, 2015	32.9	KDIGO	Cr/Diuresis	. . .	. . .	< 0.5 ml/kg/h x 6 h	Lowest Cr before hospital admission

* Cr = serum creatinine

#### Oliguria definition

Although the oliguria criteria were reported in the majority of the studies, only 10% of developed and 21.2% of developing country studies reported the frequency of oliguric patients.

The oliguria definition was stated in 81.6% (49/60) of developed country studies and in 97% (32/33) of developing country studies. In five studies from developed countries and two from developing countries, the oliguria definition was not available in the manuscript and was obtained through contact with the researcher by electronic mail. In total, 11 different definitions for oliguria were used. The most frequent was “urinary volume < 0.5 ml/kg/h for 6 h”, which was found in 81.6% (40/49) of developed and in 68.7% (22/32) of developing country studies (Tables 4 and 5 and Fig 2).

#### Reference serum creatinine definition

The reference SCr definition was available in 68.3% (41/60) of studies in developed countries and 57.5% (19/33) in developing countries. In 12 studies from developed countries and 8 from developing countries, this information was not available in the manuscript, and it was obtained through contact with the researcher. We found 29 different definitions for reference SCr (Tables 4 and 5 and Fig 2), and there was no particular dominant definition.

### Timeframe for AKI assessment

The timeframe for AKI diagnosis was available in 58.3% (35/60) of studies in developed countries and 69.6% (23/33) in developing countries. Among those reporting this information, the most used definition for timeframe was “*until ICU discharge or death*” found in 42.8% (15/35) of developed and in 39.1% (9/23) of developing country studies (Tables 4 and 5).

### Incidence and AKI etiology

The incidence of AKI was reported in 91.3% (85/93) of the analyzed studies (Tables 4 and 5). Most studies of both developed (54.7%, 29/53) and developing countries (82.1%, 23/28) reported an AKI incidence up to 40%. According to different AKI definitions, AKI incidence varied from 2.1% [12] to 78.7% [13] in developed countries and from 0.5% [14] to 65% [15] in developing countries.

AKI etiology was described in 46.6% (28/60) of studies from developed countries and in 66.6% (22/33) of studies from developing countries. Sepsis and shock were the most common causes of AKI in both developed and developing countries (see Tables 6 and 7). The frequency of sepsis as the cause of AKI in developed countries ranged from 4.4% [13] to 100% [16], and half of the studies had frequencies greater than 40% (9/18). In developing countries, the frequency of sepsis as a cause of AKI ranged from 2.9% [17] to 100% [18], and 66.7% of the studies reported a frequency greater than 40% (12/18). In developed countries, shock was less frequently reported as a cause of AKI than in developing countries. Only one study reported tropical diseases (leptospirosis) as contributing to AKI etiology (Tables 6 and 7).

**Table 6:** Characteristics of patients with AKI—developed countries.

Author	Median age	Male gender (%)	AKI etiology	Comorbidities	Patient location before ICU	Severity scores
Abosaif et al, 2005	65	68	Sepsis 41.2%	CVD* 41.2%, COPD* 41.2%, CKD* 48.4%	Surgery room 47.5%, ward 52.5%	APACHE II 22.3/ SAPS II 51.5
Bagshaw et al, 2005	65	62	Shock 15%, drugs 85%	CVD 50%, COPD 34.6%, DM* 30%, CKD 18.8%	. . .	APACHE II 33
Chawla et al, 2005	65	54	. . .	CVD 30.4%, DM 36.6%, CKD 18%	. . .	APACHE II 12.5
Ostermann et al, 2005	61	64	Surgery 59.4%, medical 40.5%	. . .	Surgery room 57.1%, ward 16.4%, emergency 15.6%	APACHE II 14
Ahlstrom et al, 2006	63		. . .	. . .	. . .	APACHE II 22
Herrera-Gutiérrez et al, 2006	59	71	Shock 36.6%, drugs 38.4%, mixed 21.2%	CVD 84.7%, COPD 18%, CKD 4.4%	Surgery room 18.1%, ward 31.3%, emergency 7.9%	APACHE II 22.5/ SOFA 11.9
Hoste et al, 2006	63	49	Sepsis 17.2%, heart failure 33%, neurological 20.7%	. . .	Surgery room 61.5%, ward 38.3%	APACHE II 56 / SOFA 7.8
Bagshaw et al, 2007	64	61	. . .	CVD 11.6%, COPD 8.5%, HD* 4.8%	Surgery room 49.6%, ward 50.4%	APACHE II 16.4
Cruz et al, 2007	64	62	Sepsis 25.6%, shock 38%, drugs 14.5%, contrast 5.6%	CVD 58.5%, DM 25.6%	Surgery room 27,8%, ward 37.2%	. . .
Eachempati et al, 2007	63	60	. . .	. . .	Surgery room 100%	APACHE III 81
Ostermann et al, 2007	61	64	. . .	. . .	Surgery room 57.1%, ward 16.4%, emergency 15.6%	APACHE II 21
Bagshaw et al, 2008	55	16	. . .	CVD 17.7%, COPD 9.7%, HD 3%	Emergency 100%	APACHE II 20.9/APACHE III 71.7
Bagshaw et al, 2008	62	60	Sepsis 27.8%	. . .	Surgery room 49.7%	APACHE II 16.9
Barrantes et al, 2008	67	57	Sepsis 19.4%, respiratory failure 35.2%	. . .	. . .	APACHE II 15
Lopes et al, 2008	59	59	. . .	CVD 53.2%	Ward 76.4%, others 23.6%	SAPS II 46.3
Ostermann et al, 2008	61	61	Surgery 45.7%, medical 54.2	. . .	Surgery room 43.1%, ward 24.2, Emergency 18.6%	APACHE II 18, SOFA 7
Abelha et al, 2009	64	65	Emergency 33%	CVD 46%	Emergency 33%	APACHE II 13, SAPS II 33
Andrikos et al, 2009	72	67	Sepsis 45.3%, shock 9.4%, Surgery 21.2%, drugs 7.1%	CVD 67.5%, DM 32.9%	Surgery room 31.8%, ward 38.8%, emergency 20%	. . .
Cartin-Ceba et al, 2009	66	54	. . .	. . .	. . .	APACHE III 11.5
Costantini et al, 2009	46	71	Trauma 100%	. . .	Emergency 100%	. . .
Joannidis et al, 2009	63	61	post surgery 34.5%, emergency 65.3%	CVD 9.1%, COPD 4.2%, DM 8.5%, HD 3.1%	Surgery room 55%, ward 42.2%	SOFA 3, SAPS III 47
Thakar et al, 2009		98	. . .	CVD 51.6%, COPD 22.1%, DM 28.6%, CKD 3.6%, HD 4.5%	Surgery room 22%, ward 45%	. . .
Aldawood et al, 2010	60	55	. . .	. . .	Surgery room 3%, ward 84%, others 13%	APACHE II 33
Cruz et al, 2010	64	69	. . .	DM 15.6%, CKD 6.6%	Surgery room 8.3%, ward 54.6%, emergency 37.1%	APACHE II 20/ SOFA 5/SAPS II 45
Elseviers et al, 2010	64	63	Shock 45.5%, drugs 54.5%	. . .	Surgery room 27.2%, ward 72.8%	APACHE II 23.9/ SOFA 9.2
Park et al, 2010	63	63	. . .	. . .	Surgery room 17.5%, ward 82.5%	SOFA 7.4
Clec'h et al, 2011	66	59	. . .	CVD 17.9%, COPD 12.9%, HD 6.3%, DM 16.4%	Surgery room 10.9%, ward 71.8%, emergency 17.3%	APACHE II 19.9/ SOFA 5.3/ SAPS II 50.2
Darmon et al, 2011	61	49	Sepsis 67.5%, shock 19.8%, Drugs 20.7%, CKD 16.3%, contrast 8.9%	. . .	. . .	SAPS II 46
Garzotto et al, 2011	66	59	CVD 12.1%, respiratory failure 27.4%, neurological 17%, trauma 14.4%	. . .	Surgery room 52.7%, ward 47.3%	APACHE 18, SOFA 5, SAPS II 43
Macedo et al, 2011	69	58	Sepsis 47%	CVD 70%, DM 30%, CKD 12%, HD 35%, COPD 2.1%	…	. . .
Macedo et al, 2011		63	Sepsis 18.5%, shock 34.7%, respiratory failure 52%, drugs 18.5%	CVD 31.7%, DM 30.5%, HD 17.3%	. . .	. . .
Mandelbaum et al, 2011	66	58	. . .	. . .	. . .	SOFA 5
Medve et al, 2011	65	56	Sepsis 44%, shock 39%, post operatory 16%, drugs 2%	. . .	Surgery room 64.3%, ward 35.7%,	SOFA 6/ SAPS II 47.5
Ostermann et al, 2011	62	63	. . .	. . .	Surgery room 38%, ward 30.7%, emergency 14.1%, others 17.2%	APACHE II 18/ SOFA 7
Piccini et al, 2011	66	59	CVD 12.1%, respiratory failure 27.4%, neurological 17%, trauma 14.4%	. . .	Surgery room 52.7%, ward 47.3%	APACHE 18, SOFA 5, SAPS II 43
Prowle et al, 2011		59	. . .	. . .	Surgery room 47.7%, ward 52.3%	. . .
Clark et al, 2012	59	66	. . .	CVD 55%, COPD 24%, HD 15%, DM 34%	Surgery room 41%, ward 45%, emergency 14%	APACHE II 27/SOFA 13.4
Han et al, 2012	68	60	Sepsis 4.4%, CVD 36.7%, Tumor 16.9%	. . .	Ward 97.9%	APACHE II 19.4
Medve et al, 2012	67		Sepsis 45.8%	. . .	Surgery room 100%	SOFA 5/ SAPS II 40
Odutayo et al, 2012	65	71	. . .	CVD 55%, DM 32%	Ward 86%, emergency 6%, Others 8%	. . .
Shashaty et al, 2012	40	74	. . .	CVD 16.8%, DM 6%	Emergency 100%	APACHE III 73
Sigurdsson et al, 2012	59	61	Sepsis 26%, shock 46.4%, respiratory failure 35.4%, surgery 35%	CVD 46%, DM 14%, COPD 25%, HD 3%	. . .	APACHE II 23
Vaara et al, 2012	63	63	. . .	. . .	Surgery room 39.7%, ward 36.7%	SOFA 10/ SAPS II 48
Wohlauer et al, 2012	37	74	. . .	. . .	Surgery room 100%	MOF 184
Allegretti et al, 2013		63	. . .	CVD 29%, DM 29%, COPD 20%, HD 18%	Surgery room 45%, ward 55%	Charlson 2
Alsultan et al, 2013	54		. . .	CVD 22%, DM 20.9%	. . .	APACHE II 29.9
Fuchs et al, 2013	63	60	. . .	CVD 26%, DM 13%, HD 5%	Surgery room 24,3%, ward 75.7%	SOFA 8.9/ SAPS I 16.3
Legrand et al, 2013	71	60	. . .	CVD 43%; DM 15%; COPD 9%; HD 7%	Surgery room 100%	SAPS II 57
Nisula et al, 2013	65	65	. . .	CVD 77.5%, COPD 10.8%, DM 22.9%, CKD 8.1%	Surgery room 39.4%; emergency 82.1%	SOFA 9/ SAPS II 42
Poukkanen et al, 2013	64	92	Sepsis 100%	CVD 19.6%, DM 5.2%	Emergency 96.7%	SOFA 9, SAPS II 43
Poukkanen et al, 2013	66	66	Sepsis 31.6%	CVD 10.6%, COPD 12%, DM 24.8%	Surgery room 24.2%, emergency 97%	SAPS II 48, SOFA 11
Doi et al, 2014	66	61	. . .	. . .	Surgery room 49.6%, ward 50.4%	APACHE II 15
Han et al, 2014	68	60	Sepsis 4.6%, CVD 30.8%, post operatory 1.9%	DM 12.2%, CKD 8.7%	. . .	APACHE II 18.4
Linder et al, 2014	61	68	Sepsis 81%, post operatory 27.8%	CVD 8.1%, COPD 12.7%, DM 2.6%	. . .	APACHE II 24.6
Shinjo et al, 2014	63	66	. . .	CVD 38.5%, DM 18.4%, COPD 3.5%, HD 3.1%	Surgery room 74.3%, ward 13.2%; emergency 12.5%	APACHE II 9/SOFA 4/ SAPS II 28
Udy et al, 2014	54	63	. . .	. . .	Surgery room 44.8%, ward 9.3%; emergency 45.9%	IQR 3
Bouchard et al, 2015	62	62	. . .	. . .	. . .	. . .
Harris et al, 2015	59	59	. . .	CVD 46%, DM 26%	Surgery room 100%	APACHE III 66
Rimes-Stigiare et al, 2015	68	60	. . .	. . .	. . .	APACHE II 25/ SAPS II 55
Vanmassenhove et al, 2015	66	67	Sepsis 100%	. . .	. . .	APACHE II 27

Neurologic etiologies of AKI include polyuria of diabetes insipidus and salt wasting syndrome; CKD refers to AKI on baseline CKD patients. Comorbidities: CVD = cardiovascular disease DM = diabetes mellitus; COPD = chronic obstructive pulmonary disease; HD = hepatic disease; CKD = chronic kidney disease

**Table 7:** Characteristics of patients with AKI—developing countries.

Author	Median age	Male gender (%)	AKI etiology	Comorbidities	Patient location before ICU	Severity scores
Mataloun et al, 2006	55	46	. . .	CVD 35.7%, DM 14%	Surgery room 44.3%; ward 27.6%; emergency 28.1%	APACHE II 15.2
Silva Junior et al, 2006	50	62	Sepsis 40.6%, shock 48.4%, drugs 21.9%	CVD 25.8%, COPD 28.9%	. . .	. . .
Chow et al, 2007	58		Sepsis 41%, shock 43.6%	. . .	Surgery room 30.8%, ward 69.2%; obstetrics1%	. . .
Daher et al, 2008	45	77	Sepsis 41.5%, shock 40.2%, drugs 10.2%	CVD 13%, COPD 19%	Clinical ward 100%	APACHE II 28
Lima et al, 2008	45	77	Infection 100%	CVD 14.2%, HD 40.1%	. . .	APACHE II 27
Fernandes et al, 2009	56	62	Sepsis 63%	CVD 63%, HD 22%	Surgery room 44%, ward 51.5%, emergency 45%	APACHE II 25.5
Friedericksen et al, 2009	44	61	Sepsis 50%, multiple organ failure 78%	CVD 21.7%, DM 19%	. . .	APACHE II 23.4
Chang et al, 2010	62	70	Sepsis 55%	DM 27.5%, HD 42%	. . .	APACHE II 22.8/ SOFA 9.19
Maccariello et al, 2010	70		Sepsis 74%, shock 75%, contrast 33%	CVD 68%, DM 28%	Surgery room 19%, ward 13%; emergency 68%	SOFA 8.3/ SAPS III 70
Balushi et al, 2011	61	60	Shock 53.6%, drugs 46.4%	CVD 53.7%, DM 59.9%	. . .	. . .
Ponce et al, 2011	57	56	. . .	. . .	. . .	. . .
Fonseca Ruiz et al, 2011	53	53	Sepsis 19.6%	CVD 44,6%, DM 18,2%	Surgery room 38.2%, ward 57.6%, emergency 4.2%	APACHE II 13/SOFA 4/ SAPS II 27
Samimagham et al, 2011	40	71	. . .	. . .	Surgery room 52.3%, ward 11.1%	APACHE II 23.9
Alves et al, 2012	50	45	Sepsis 65%	. . .	Surgery room 27.9%, ward 67.4% Obstetrics 4.7%	SOFA 9.8
Chen et al, 2012	69	75	. . .	CVD 73%, DM 61%	Ward 100%	APACHE II 14
Daher et al, 2012	55		Sepsis 40%, drugs 14%, leptospirosis 12%	CVD 24,7%; DM 14,8%	Ward 100%	. . .
Lai et al, 2012	64	66	. . .	CVD 58.4%, DM 28.7%	Surgery room 70.7%, emergency 29.3%	. . .
Wahrhaftig et al, 2012	66	47	Sepsis 74.2%, shock 17%	CVD and DM 99%	Surgery room 13.8%, ward 29.7%, emergency 17.3%, others 35.6%	APACHEII 13 /SOFA 3
Zhou et al, 2012	59	69	Sepsis 30.6%, respiratory failure 79.9%	CVD 36%, DM 12.8%	Surgery room 3.7%, ward 79.1%, emergency 7.6%, other 9.6%	APACHE III 45.4 / SOFA 5.1
Dalboni et al, 2013	67	66	. . .	CVD 39%, DM 18%	. . .	APACHE II 20
Levi et al, 2013	64	45	Sepsis 46.8%, CVD 42%	CVD 46.8%, DM 32.2%	Surgery room 30.5%, ward 30.5%, emergency 22.6%	APACHE II 15
Silva et al, 2013	57	60	. . .	COPD 77,3%, HD 49,2%	. . .	SAPS III 69.7
Singh et al, 2013	51	0	Sepsis 35.2%, shock 14.2%, drugs 23.5%	CVD 13,7%, COPD 23,5%	. . .	. . .
Daher et al, 2014	46	72	. . .	HIV/AIDS 30%, Tuberculosis 12%	Ward 100%	APACHE II 50
Luo et al, 2014	61	65	Sepsis 32.2	CVD 5.9%, COPD 6.1%, CKD* 6.1%, DM 18.9%,	Surgery room 57%,	SOFA 6
Morales-Buenrostro et al, 2014	52	54	. . .	CVD 16.2%, DM 24.3%	Surgery room 21.6%, ward 64,9%, others 10.8%	. . .
Peng et al, 2014	52	68	Sepsis 100%	. . .	. . .	APACHE II 20.8
Wijewickrama et al, 2014	48	62	Sepsis 28.7%, shock 12.1%	CVD 28%, DM 27%	. . .	SOFA 9
Bentata et al, 2015	28		Obstetrics 100%	CVD 19.5%,	Emergency 22%	. . .
Bouchard et al, 2015	59	63	. . .	. . .	. . .	. . .
Heegard et al, 2015	26	98	Trauma 100%	. . .	Emergency 100%	. . .
Ralib et al, 2015	50	64	. . .	CVD 36.4%, DM 28.7%	Surgery room 25.2%, ward 74.8%	APACHE II 19.4/ SOFA 8.7
Santos et al, 2015	43	66	Sepsis 2.9%, CVD 3.2%, emergency 48.4%	DM 12.9%	Emergency 48.8%	APACHE II 10

Comorbidities: CVD = cardiovascular disease; DM = diabetes mellitus; COPD = chronic obstructive pulmonary disease; HD = hepatic disease; CKD = chronic kidney disease

### Patient characteristics

#### Age

Almost 40% (22/56) of the studies in developed countries described a mean age above 65 years in AKI patients (ranging from 37 [12] to 72 years [19]), while only 12.1% (4/33) of the studies in developing countries reported an age higher than 65 years in AKI patients (ranging from 26 [20] to 70 years [21]) (Tables 6 and 7). The weighted mean ages were 62.0 and 56.8 years for developed and developing country patients, respectively.

#### Gender and ethnicity

Male sex was predominant in AKI patients in both groups of countries: 59.6% (34/57) and 67.8% (19/28) of the studies in developed and developing countries, respectively, reported a male frequency above 60%. The weighted male frequencies were 67.1% and 64.5% for developed and developing country patients, respectively.

Only 9.5% (9) of the studies reported the patients’ ethnic background (Tables 6 and 7).

#### Comorbidities

Comorbidities were assessed in 51.6% (31/60) and 78.7% (26/33) of studies from developed and developing countries, respectively. The most prevalent comorbidities were cardiovascular diseases (CVD), diabetes and chronic respiratory disease.

In developed counties, a CVD frequency greater than 40% was reported in 51.6% (16/31), versus 30.4% (7/23) in developing countries. The weighted CVD frequencies were 41.3% and 32.6% for developed and developing country patients, respectively.

The frequency of diabetes was similar in both groups of countries. In approximately 60% (16/27 in studies of developed and 10/18 of developing countries) of studies, the prevalence of diabetes was over 20% in the studied population (Tables 6 and 7). The weighted diabetes frequencies were 27.3% and 24.5% for developed and developing country patients, respectively.

#### Severity scores

The most reported severity scores were APACHE II and SOFA. The Apache II score in AKI patients was reported in 32 and 18 studies from developed and developing countries, respectively. The APACHE II score had a similar distribution in the two groups of countries, ranging from 9 [22] to 56 [23] in developed country studies and from 10 [17] to 50 [24] in studies from developing countries. Approximately half of the studies had an APACHE II score up to 20 (16/32) in developed and in developing countries (9/18) (Tables 6 and 7). The weighted APACHE II scores were 18.7 and 21.0 for developed and developing country patients, respectively.

The SOFA score was reported in 21 and 10 studies from developed and developing countries, respectively. The distribution of the SOFA score was similar between groups, ranging from 3 [25] to 13.4 [26] in studies from developed countries and from 3 [27] to 9.8 [28] in developing country studies. In studies where this information was available, a SOFA score over 5 was reported by 71% (15/21) of studies from developed countries and 80% (8/10) of studies in developing countries (Tables 6 and 7). The weighted SOFA scores were 7.6 and 8.2 for developed and developing country patients, respectively.

#### Patient location before ICU

Most of the AKI patients who were admitted to the ICU came from surgical and clinical wards units. The majority of manuscripts from both developed countries (13/16) and developing countries (9/11) reported that up to 50% of patients with AKI had hospital admission in emergency situations (Tables 6 and 7).

### Outcomes

#### Length of ICU and hospital stay

In developed and in developing countries, 71.6% (43/60) and 69.6% (23/33) of the studies reported the length of ICU stay for AKI patients, which ranged from 1 to 22 days and from 5 to 23 days, respectively. The reported ICU stay was longer than seven days in 38.6% (17/44) and 80% (20/25) of developed and developing country studies, respectively (Tables 8 and 9). The weighted mean ICU stay lengths were 7.2 and 12.2 days for developed and developing country patients, respectively.

**Table 8:** Outcomes—Developed countries.

Autor	ICU stay (days)	Hospital stay (days)	RRT in ICU AKI patients (%)	Type of RRT	Mortality in ICU AKI patients (%)
Abosaif et al, 2005	. . .	. . .	38.8	C 100%	47.5
Bagshaw et al, 2005	8.1	22	6	C 61%, I 20%	50
Chawla et al, 2005	. . .	. . .	37.1	. . .	
Ostermann et al, 2005	. . .	. . .	. . .	C 92%, I 7.1%	66.7
Ahlstrom et al, 2006	. . .	. . .	7	. . .	16.6
Herrera-Gutiérrez et al, 2006	15.6	19.9	38	. . .	46.8
Hoste et al, 2006	3	16	6	. . .	
Bagshaw et al, 2007	4.4	14.2	. . .	. . .	
Cruz et al, 2007	10	. . .	30.3	C 50.7%, I 14.1%	36.3
Eachempati et al, 2007	16	15.9	19.8	I 100%	45
Ostermann et al, 2007	. . .	. . .	12.2	C 80.2%, I 5.2%	28.4
Bagshaw et al, 2008	. . .	. . .	. . .	. . .	16.7
Bagshaw et al, 2008	3.7	14.6	. . .	. . .	24.2
Barrantes et al, 2008	3	9	15	. . .	
Lopes et al, 2008	8.2	. . .	27.2	. . .	41.3
Ostermann et al, 2008	7	. . .	23	C 93.6%, I 0.4%	31.1
Abelha et al, 2009	2.8	25	. . .	. . .	17.2
Andrikos et al, 2009	13	. . .	53.5	C 86%, I 13.2%	64.7
Cartin-Ceba et al, 2009	. . .	. . .	19	C 39%, I 61%	20
Costantini et al, 2009	13.6	25	7	. . .	15.9
Joannidis et al, 2009	2.8	. . .	. . .	. . .	16
Thakar et al, 2009	. . .	. . .	4.4	. . .	
Aldawood et al, 2010	15	. . .	9	. . .	64
Cruz et al, 2010	7	. . .	. . .	. . .	17.3
Elseviers et al, 2010	16.4	34.2	49.9	C 42%, I 58%	58
Park et al, 2010	. . .	17.2	. . .	. . .	62.8
Clec'h et al, 2011	7	. . .	19	. . .	
Darmon et al, 2011	. . .	. . .	22.1	. . .	36.7
Garzotto et al, 2011	5	. . .	8	. . .	21.7
Macedo et al, 2011	4	8	6	. . .	
Macedo et al, 2011	3	8	. . .	. . .	9.5
Mandelbaum et al, 2011	7	16	. . .	. . .	12.4
Medve et al, 2011	4.5	13.5	15.1	I 64.8%	39.3
Ostermann et al, 2011	7	. . .	. . .	. . .	31.1
Piccini et al, 2011	5	. . .	8	. . .	21.7
Prowle et al, 2011	. . .	. . .	39	. . .	22.5
Clark et al, 2012	. . .	. . .	. . .	C 77%, I 17%	
Han et al, 2012	7	. . .	17	. . .	
Medve et al, 2012	6	18	. . .	. . .	33.3
Odutayo et al, 2012	. . .	. . .	11.8	. . .	
Shashaty et al, 2012	. . .	. . .	. . .	. . .	14.9
Sigurdsson et al, 2012	17	. . .	17	. . .	9
Vaara et al, 2012	12.5	. . .	6.8	. . .	29.1
Wohlauer et al, 2012	14	. . .	23	. . .	
Allegretti et al, 2013	21	. . .	. . .	. . .	60.7
Alsultan et al, 2013	. . .	. . .	12	. . .	
Fuchs et al, 2013	. . .	. . .	. . .	. . .	. . .
Legrand et al, 2013	9	. . .	. . .	. . .	23
Nisula et al, 2013	2.8	9	10.3	. . .	. . .
Poukkanen et al, 2013	5.7	16	8	. . .	. . .
Poukkanen et al, 2013	4.2	14	. . .	. . .	. . .
Doi et al, 2014	5	. . .	. . .	. . .	12
Han et al, 2014	22	. . .	38.4	. . .	67.4
Linder et al, 2014	9.1	23.4	. . .	. . .	67.1
Shinjo et al, 2014	1	31	. . .	. . .	7.1
Udy et al, 2014	5	. . .	. . .	. . .	14
Bouchard et al, 2015	5	11	15.5	. . .	27.6
Harris et al, 2015	4.5	19	12	. . .	33
Rimes-Stigiare et al, 2015	. . .	. . .	6	. . .	35
Vanmassenhoe et al, 2015	12	. . .	13.8	. . .	23.1

RRT: renal replacement therapy; C = continuous RRT, I = intermittent RRT

**Table 9:** Outcomes in developing countries.

Autor	ICU stay (days)	Hospital stay (days)	RRT in ICU AKI patients (%)	Type of dialysis	Mortality in ICU AKI patients (%)
Mataloun et al, 2006	16.1	. . .	23.8	I 100%	76.2
Silva Junior et al, 2006	17	. . .	32	I 100%	62.5
Chow et al, 2007	13.7	. . .	16.7	P 69.2%, I 15.3%	. . .
Daher et al, 2008	11	. . .	35	I 100%	66.6
Lima et al, 2008	12	. . .	35	. . .	64.6
Fernandes et al, 2009	22	22.3	48	C 30%, I 10%	85
Friedericksen et al, 2009	10.8	. . .	17.3	C 25%, I 75%	47.8
Chang et al, 2010	11.4	. . .	. . .	. . .	60.8
Maccariello et al, 2010	23	29	. . .	C 84%	63
Balushi et al, 2011	. . .	. . .	60.7	. . .	
Ponce et al, 2011	12.9	. . .	. . .	. . .	62.5
Fonseca Ruiz et al, 2011	8.4	. . .	12.4	C 30.8%, I 69.2%	25.4
Samimagham et al, 2011	10.6	. . .	. . .	. . .	72.6
Alves et al, 2012	10.7	. . .	2.3	I 100%	29
Chen et al, 2012	. . .	. . .	. . .	. . .	
Daher et al, 2012	. . .	. . .	68	I 100%	33.6
Lai et al, 2012	. . .	. . .	. . .	. . .	. . .
Wahrhaftig et al, 2012	12	. . .		. . .	53.3
Zhou et al, 2012	11.6	. . .	63.5	. . .	50
Dalboni et al, 2013	. . .	. . .	. . .	. . .	9
Levi et al, 2013	. . .	. . .	. . .	. . .	63.1
Silva et al, 2013	17	20.5	28.7	C 85.3%, I 14.7%	78.6
Singh et al, 2013	. . .	. . .	20.6	. . .	73.5
Daher et al, 2014	. . .	. . .	27.6	. . .	62.8
Luo et al, 2014	5	27.4	. . .	. . .	. . .
Morales-Buenrostro et al, 2014	. . .	. . .	. . .	. . .	. . .
Peng et al, 2014	8.5	15.7	. . .	. . .	. . .
Wijewickrama et al, 2014	11.6	. . .	58.4	I 97.3%; P 2.7%	52.3
Bentata et al, 2015	6.5	. . .	. . .	. . .	10.2
Bouchard et al, 2015	6	10	30.2	. . .	. . .
Heegard et al, 2015	. . .	. . .	5.6	. . .	21.7
Ralib et al, 2015	6.4	14.2	25	C 36%, I 58.3%	91
Santos et al, 2015	9.5	. . .	71.7	. . .	33.3

RRT: renal replacement therapy; C = continuous RRT, I = intermittent RRT; P = peritoneal dialysis

In non-AKI patients, ICU stays longer than 7 days were not reported in developed country studies (0/24) but occurred in 44% (4/9) of studies in developing countries. In developed and developing country studies, 38.3% (23/60) and 21.2% (7/33) of the studies reported the length of hospital stay, which ranged from 8 to 31 days and 10 to 29 days, respectively. Hospital stays were longer than 15 days in 58.3% (14/24) and 66.7% (6/9) of developed and developing country studies, respectively (Tables 8 and 9). In non-AKI patients, the hospital stay was longer than 15 days in 25% (4/16) and 33.3% (1/3) in developed and in developing country studies, respectively. The weighted mean lengths of hospital stay were 15.5 and 23.6 days for developed and developing country patients, respectively.

#### Renal replacement therapy

Sixty-three percent of the analyzed studies reported the use of renal replacement therapy (RRT) in ICU AKI patients. In developed countries, 21% of the 38 studies with available data referred to the use of RRT in ICU AKI patients as being greater than 30%. In developing countries, 48% of the 21 studies with available data showed that the frequency of RRT use was higher than 30% in ICU AKI patients (Tables 8 and 9). The weighted frequencies of RRT were 8.8% and 23.8% for developed and developing country patients, respectively.

#### Mortality

Reported ICU mortality in AKI patients was greater in developing country studies. AKI mortality greater than 60% was reported in 15.9% (7/44) of studies from developed countries and 56% from developing countries (14/25) (Tables 8 and 9). The weighted frequencies of mortality were 30.8% and 54.8% for developed and developing country patients, respectively.

### Synthesis of AKI incidence

**Fig 3:**
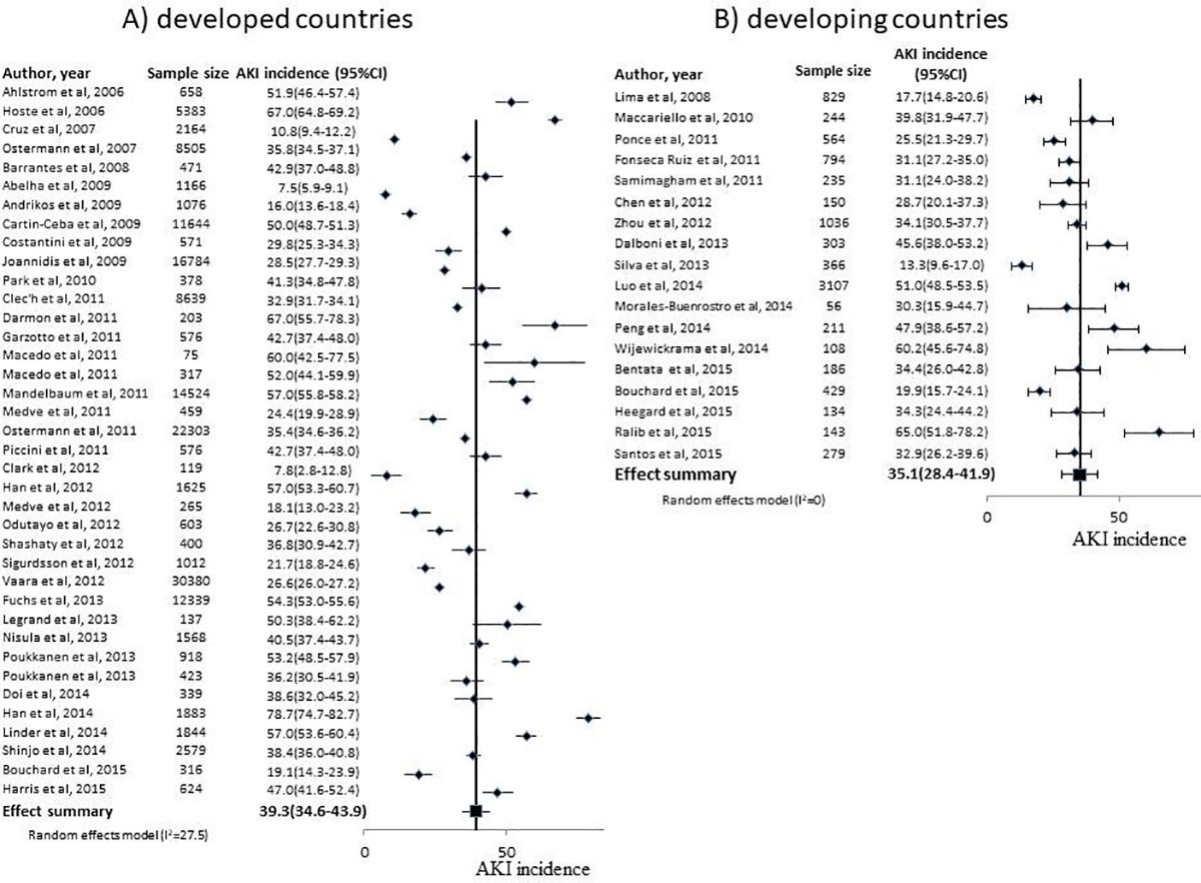
Forest plot of AKI incidence. Footnote: The studies shown are those that used the RIFLE, AKIN or KDIGO criteria for AKI definition. A) Developed country studies; B) Developing country studies.

Pooled AKI incidence estimates for developed and developing countries in the meta-analysis are presented in Table 10, according to the AKI definition used. The RIFLE, AKIN or KDIGO criteria for AKI definition was used, as defined, by 39 and 21 studies in developed and developing country studies, respectively. One study in developed countries and 3 in developing countries studies did not report AKI incidence, so 38 and 18 studies, respectively, had an AKI incidence estimation included in the meta-analysis. The pooled estimate of AKI incidence in developed and developing countries is shown in Fig 3. There was a tendency towards a greater incidence in developed countries, although this was not significant. When only prospective studies were analyzed, this tendency disappeared (Table 10).

**Table 10:** Pooled AKI frequency of developed and developing countries’ studies according to AKI Definition.

Subgroup	Country group	Studies	Patients	AKI frequency	95% confidence	Test for heterogeneity	
		(n)	(n)	(%)	interval	I^2^ Index	*Q* Test p-value
RIFLE/AKIN/KDIGO	Developed	38	153,846	39.3	34.6–43.9	27.5	0.062
	Developing	18	9,174	35.1	28.4–41.9	-15.1	0.612
RIFLE	Developed	14	71,954	37.4	30.0–44.8	13.0	0.018
	Developing	4	1,742	26.9	15.3–42.0	26.9	0.250
AKIN	Developed	18	72,677	36.9	29.5–44.3	22.7	0.185
	Developing	8	3,372	30.8	25.5–36.1	42.2	0.097
KDIGO	Developed	6	9,215	50.7	38.3–63.1	7.26	0.370
	Developing	6	4,060	43.8	34.7–52.8	15.2	0.316
RIFLE/AKIN/KDIGO–Prospective studies	Developed	18	29,164	37.4	30.9–43.9	26.4	0.145
	Developing	13	6,677	36.2	27.4–44.9	-16.2	0.587

*Included the studies that used the RIFLE, AKIN or KDIGO definition

**Fig 4:**
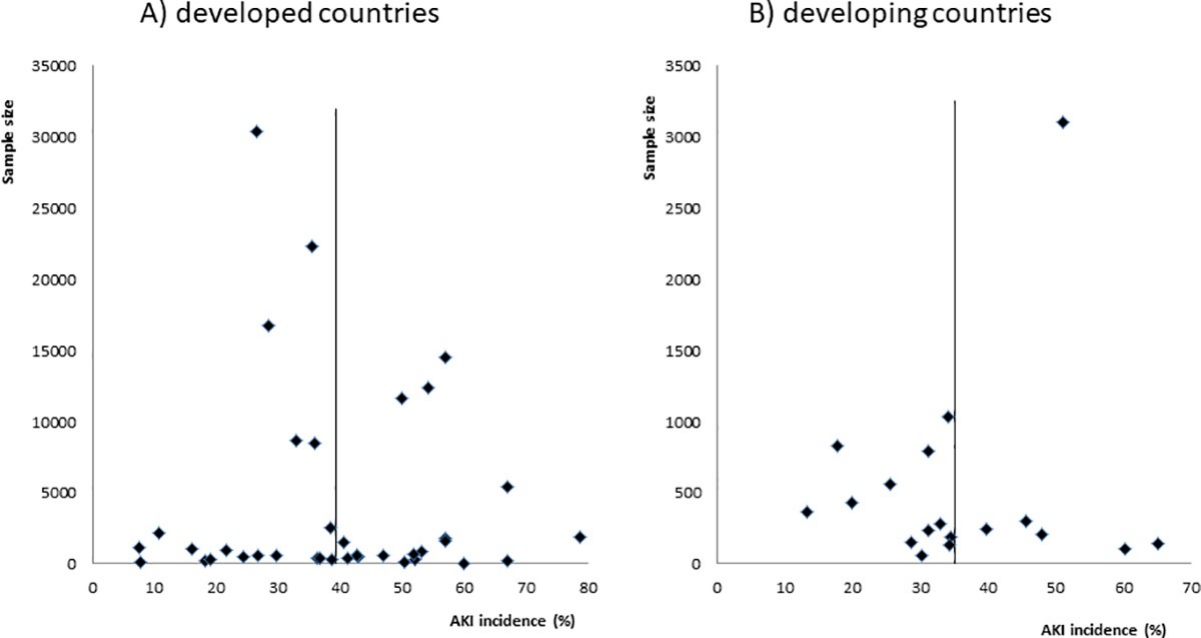
Funnel plot of sample size of studies as a function of AKI incidence. Footnote: The studies shown are those that used the RIFLE, AKIN or KDIGO criteria for AKI definition. A) Developed country studies; B) Developing country studies. Pooled AKI incidence is depicted as a vertical line.

Fig 4 shows the funnel plot for both country groups in which individual study frequency of AKI is a function of their sample size with the pooled incidence of studies that used the RIFLE, AKIN or KDIGO criteria for the AKI definition being depicted as a black line. Note that the Fig 4A (developed countries) had a 10-fold greater sample size than Fig 4B (developing countries). The studies with a greater sample size depart from the polled estimated AKI incidence, suggesting the possibility of publication bias or bias resulting from the lack of standardizing reference creatinine, oliguria, and the timeframe for AKI assessment.

## Discussion

We found a high incidence of AKI in both country categories, and a tendency towards a greater incidence in developed countries. Due to the differences in AKI definitions, timeframe and the types of studied population, the reported incidence varied from 0.5% to 78%.

Our review covered a 10-year period. Thus, different AKI definitions were used for AKI assessment, including the RIFLE, AKIN and KDIGO criteria [29,30,31]. Only two-thirds of the studies reported the definition for reference serum creatinine, with 29 different definitions used, which results in high heterogeneity of AKI incidence estimate [32]. In the most recent AKI definitions (RIFLE, AKIN and KDIGO), the reference SCr is the value observed up to seven days or 48 hours before the SCr increase defining the AKI diagnosis. However, we observed that several studies used as reference SCr values obtained months, or even one year before the AKI episode, which is not consistent with the current AKI definitions. When the reference serum creatinine was not available, the MDRD formula has been used for estimation of the missing SCr value, which is a flawed methodology as it can misdiagnose AKI in CKD patients [12,33,34]. The oliguria definition was more uniform, with recent studies correctly using the RIFLE, AKIN and KDIGO oliguria definitions. The addition of urine output criteria was associated with higher and earlier AKI detection and incidences in critically ill patients [32].

Another important caveat to create a valid comparison between developed and developing countries is the striking differences in the number of studies and the sample sizes. Eighty per cent of the world population lives in developing countries, but only one-third of the studies sample reported data from them, with the majority assessing a single center with a relatively small number of patients [35]. Moreover, approximately half of the studies from developing countries were from Brazil, and only two were from Africa. On the other hand, approximately 40% of developed country studies assessed more than five centers. The sample size from developed countries studies was more than 30-fold greater compared to those from developing countries. There is a clear underrepresentation of developing countries that is probably caused by a lack of health resources and electronic medical records, as well as difficulty in gathering epidemiological data and, consequently, conducting adequate large observational studies. These studies can be more capable to determine the true burden of a disease than trials and more valuable in assessing the incidence and prevalence of the disease [36]. A snapshot of worldwide AKI incidence found more severe AKI presentation in patients from developing countries, which was considered to be due to delay in AKI recognition and treatment, adversely affecting the outcomes [7,37].

The incidence of AKI development in the ICUs was similar in both types of countries, with a numeric tendency to be greater in developed country studies. When only prospective studies were analysed, this tendency disappeared. Developing and developed countries have very distinct healthcare patterns. In developing countries, deficiencies in health structure, long distance from the community to the hospital and poor transportation systems limit patient access to healthcare. Lack of universal health coverage and insufficient funding for the health system imposes significant cost of treatment for the patients and family, including high cost procedures such as ICU and renal replacement therapy [38]. Tropical infectious diseases, animal venoms, natural medicine, abortion and eclampsia are known to be important AKI etiological factors in developing countries [39,40]; however, their incidence was extremely low in the ICU population. This is likely due to the limited number of ICUs, which are located mostly in larger urban cities, as well as inadequate recognition of high-risk AKI patients in the primary health system. Furthermore, difficulty transporting patients due to geographical and economic issues may contribute to this situation [39]. As a consequence, developing country’s ICUs reflect tertiary hospitals and university hospitals mostly from an urban population. Thus, patient characteristics were similar in both types of country. Sepsis and shock were the main causes of AKI in both developed and developing countries, but the frequency of sepsis was approximately 50% greater in developing country studies. In developed country studies, AKI patients were older, which likely reflects higher population longevity, better socioeconomic conditions and more structured health services. Overall, cardiovascular diseases were the most frequently reported comorbidity, although they were more frequent in developed country studies.

AKI was associated with poor outcomes, higher length of stay (LOS) and mortality, which is consistent with other studies [41,42]. In developing country studies, AKI had higher LOS and mortality compared to developed countries, although patients were younger, had less CVD and had similar APACHE II scores. Difficulty accessing health services [39,43] and lack of infrastructure, including ICU beds and human resources for care of the critically ill in these countries [44,45], are probably the cause of worse outcomes in such a low resource setting. It is possible that the patients treated in developing countries are transferred to an ICU at a late stage of disease progression and have a reduced change of recovering [45]. The finding of higher frequency RRT use in developing countries supports this hypothesis.

This systematic review highlighted important caveats for the comparison between ICU AKI epidemiology in developed and developing countries. The vast majority of studies assessed university tertiary hospitals, limiting the generalizability of the results. Different AKI definitions were used over time, and even when the new AKI criteria were used, there is an important lack of standardization for reference serum creatinine. Most of the studies from developing countries were single center. The number of patients and ICUs assessed in developed country studies was greater than 30-fold and 10-fold higher than in developing countries, respectively, highlighting the underrepresentation of developing countries.

## Conclusion

AKI incidence was high in both types of countries. Patient characteristics were mostly similar, but outcomes were worse for patients in developing country studies. Despite patient´s similarities, the non-inclusion of secondary hospitals and the differences in the number of studies and sample sizes exemplify the challenge of comparing developing and developed country AKI epidemiology. The widespread application of AKI definitions has made it possible to compare AKI epidemiology across different settings. However, an effort to standardize reference serum creatinine, oliguria and the timeframe for AKI assessment is crucial. There is an urgent need for larger, prospective, multicenter studies that assess broader populations from developing countries.

## Supporting information

S1 File Supplementary material search strategies. (DOCX)

S2 File Supplementary material syst review references. (DOCX)

S3 File PRISMA checklist. (DOCX)
