# Supplementary material for: A systematic review and meta-analysis of acute kidney injury in the intensive care units of developed and developing countries
Source: PLoS One. 2020 Jan 17;15(1):e0226325. doi: 10.1371/journal.pone.0226325 (PMC6968869; doi:10.1371/journal.pone.0226325)
Supplement: S1 File — (DOCX) [file pone.0226325.s001.docx]

| #1 | Search **(((((acute kidney injury) OR acute kidney insuficiency) OR acute kidney failure) OR acute renal injury) OR acute kidney insuficiency) OR acute kidney failure** | 57181 |
| --- | --- | --- |
| #2 | Search **(((intensive care) OR intensive care unit) OR critical ill) OR critical patient** | 394151 |
| #1 and #2 | Search (((((((acute kidney injury) OR acute kidney insuficiency) OR acute kidney failure) OR acute renal injury) OR acute kidney insuficiency) OR acute kidney failure)) AND ((((intensive care) OR intensive care unit) OR critical ill) OR critical patient) | 5635 |
| #4 | Search **(((((((acute kidney injury) OR acute kidney insuficiency) OR acute kidney failure) OR acute renal injury) OR acute kidney insuficiency) OR acute kidney failure)) AND ((((intensive care) OR intensive care unit) OR critical ill) OR critical patient)** Sort by: **PublicationDate** Filters: **Publication date from 2005/07/01 to 2015/07/31; Humans; Adult: 19+ years** | 2042 |
|  |  |  |

**Search strategies**

PUBMED

CENTRAL (Cochrane Controlled Register of Trials)

| #1 | acute kidney injury or "acute kidney failure" or "acute renal isuficiency" or "acute kidney insuficiency" or "acute renal injury" or "acute renal failure" | 2225 |
| --- | --- | --- |
| #2 | intensive care unit or "intensive care" or "critical ill patient" or "critical ill" or "intensive care units" | 19270 |
| #3 | #1 and #2 -Online Publication Date from Jul 2005 to Jul 2015 | 74 |

LILACS

| # 1 | (tw:(acute kidney injury)) OR (tw:(acute kidney failure)) OR (tw:(acute kidney insuficiency)) OR (tw:(acute renal injury)) OR (tw:(acute renal failure)) OR (tw:(acute renal insuficiecy)) | 700 |
| --- | --- | --- |
| # 2 | (tw:(intensive care)) OR (tw:(intensive care unit)) OR (tw:(critical ill)) OR (tw:(critical ill patient)) | 4900 |
| # 3 | #1 and #2 | 132 |

IBEC

| # 1 | (tw:(acute kidney injury)) OR (tw:(acute kidney failure)) OR (tw:(acute kidney insuficiency)) OR (tw:(acute renal injury)) OR (tw:(acute renal failure)) OR (tw:(acute renal insuficiecy)) | 456 |
| --- | --- | --- |
| # 2 | (tw:(intensive care)) OR (tw:(intensive care unit)) OR (tw:(critical ill)) OR (tw:(critical ill patient)) | 2027 |
| # 3 | #1 and #2 | 62 |
