# Supplementary material for: A systematic review and meta-analysis of acute kidney injury in the intensive care units of developed and developing countries
Source: PLoS One. 2020 Jan 17;15(1):e0226325. doi: 10.1371/journal.pone.0226325 (PMC6968869; doi:10.1371/journal.pone.0226325)
Supplement: S2 File — (DOCX) [file pone.0226325.s002.docx]

**Systematic review references**

ABOSAIF, N. Y. et al. The outcome of acute renal failure in the intensive care unit according to RIFLE: model application, sensitivity, and predictability. **Am J Kidney Dis,** v. 46, n. 6, p. 1038-48, Dec 2005. ISSN 0272-6386.

BAGSHAW, S. M.; GEORGE, C.; BELLOMO, R. Prognosis for long-term survival and renal recovery in critically ill patients with severe acute renal failure: a population-based study. **Crit Care,** v. 9, n. 6, p. R700-9, 2005. ISSN 1364-8535.

CHAWLA, L. S. et al. Identifying critically ill patients at high risk for developing acute renal failure: a pilot study. **Kidney Int,** v. 68, n. 5, p. 2274-80, Nov 2005. ISSN 0085-2538.

OSTERMANN, M. E.; CHANG, R. W. Prognosis of acute renal failure: an evaluation of proposed consensus criteria. **Intensive Care Med,** v. 31, n. 2, p. 250-6, Feb 2005. ISSN 0342-4642.

AHLSTROM, A. et al. Comparison of 2 acute renal failure severity scores to general scoring systems in the critically ill. **Am J Kidney Dis,** v. 48, n. 2, p. 262-8, Aug 2006. ISSN 0272-6386.

HERRERA-GUTIÉRREZ, M. E. et al. Epidemiología del fracaso renal agudo en las UCI españolas: Estudio prospectivo multicéntrico FRAMI. **Medicina Intensiva,** v. 30, p. 260-267, 2006. ISSN 0210-5691.

HOSTE, E. A. et al. RIFLE criteria for acute kidney injury are associated with hospital mortality in critically ill patients: a cohort analysis. **Crit Care,** v. 10, n. 3, p. R73, 2006. ISSN 1364-8535.

BAGSHAW, S. M.; GEORGE, C.; BELLOMO, R. Changes in the incidence and outcome for early acute kidney injury in a cohort of Australian intensive care units. **Crit Care,** v. 11, n. 3, p. R68, 2007. ISSN 1364-8535.

CRUZ, D. N. et al. North East Italian Prospective Hospital Renal Outcome Survey on Acute Kidney Injury (NEiPHROS-AKI): targeting the problem with the RIFLE Criteria. **Clin J Am Soc Nephrol,** v. 2, n. 3, p. 418-25, May 2007. ISSN 1555-9041.

EACHEMPATI, S. R. et al. Acute renal failure in critically ill surgical patients: persistent lethality despite new modes of renal replacement therapy. **J Trauma,** v. 63, n. 5, p. 987-93, Nov 2007. ISSN 0022-5282.

OSTERMANN, M.; CHANG, R. W. Acute kidney injury in the intensive care unit according to RIFLE. **Crit Care Med,** v. 35, n. 8, p. 1837-43; quiz 1852, Aug 2007. ISSN 0090-3493.

BAGSHAW, S. M. et al. A multi-center evaluation of early acute kidney injury in critically ill trauma patients. **Ren Fail,** v. 30, n. 6, p. 581-9, 2008. ISSN 0886-022x.

BAGSHAW, S. M. et al.. A comparison of the RIFLE and AKIN criteria for acute kidney injury in critically ill patients. **Nephrol Dial Transplant,** v. 23, n. 5, p. 1569-74, May 2008. ISSN 0931-0509.

BARRANTES, F. et al. Acute kidney injury criteria predict outcomes of critically ill patients. **Crit Care Med,** v. 36, n. 5, p. 1397-403, May 2008. ISSN 0090-3493.

LOPES, J. A. et al. Acute kidney injury in intensive care unit patients: a comparison between the RIFLE and the Acute Kidney Injury Network classifications. **Crit Care,** v. 12, n. 4, p. R110, 2008. ISSN 1364-8535.

OSTERMANN, M.; CHANG, R. Correlation between the AKI classification and outcome. **Crit Care,** v. 12, n. 6, p. R144, 2008. ISSN 1364-8535.

ABELHA, F. J. et al. Determinants of postoperative acute kidney injury. In: (Ed.). **Crit Care**, v.13, 2009. p.R79. ISBN 1364-8535.

ANDRIKOS, E. et al. Epidemiology of acute renal failure in ICUs: a multi-center prospective study. **Blood Purif,** v. 28, n. 3, p. 239-44, 2009. ISSN 0253-5068.

CARTIN-CEBA, R. et al. Evaluation of "Loss" and "End stage renal disease" after acute kidney injury defined by the Risk, Injury, Failure, Loss and ESRD classification in critically ill patients. **Intensive Care Med,** v. 35, n. 12, p. 2087-95, Dec 2009. ISSN 0342-4642.

COSTANTINI, T. W. et al. Redefining renal dysfunction in trauma: implementation of the Acute Kidney Injury Network staging system. **J Trauma,** v. 67, n. 2, p. 283-7; discussion 287-8, Aug 2009. ISSN 0022-5282.

JOANNIDIS, M. et al. Acute kidney injury in critically ill patients classified by AKIN versus RIFLE using the SAPS 3 database. **Intensive Care Med,** v. 35, n. 10, p. 1692-702, Oct 2009. ISSN 0342-4642.

THAKAR, C. V. et al. Incidence and outcomes of acute kidney injury in intensive care units: a Veterans Administration study. **Crit Care Med,** v. 37, n. 9, p. 2552-8, Sep 2009. ISSN 0090-3493.

ALDAWOOD, A. Outcome and prognostic factors of critically ill patients with acute renal failure requiring continuous renal replacement therapy. **Saudi J Kidney Dis Transpl,** v. 21, n. 6, p. 1106-10, Nov 2010. ISSN 1319-2442 (Print)1319-2442.

CRUZ, D. N. et al. Plasma neutrophil gelatinase-associated lipocalin is an early biomarker for acute kidney injury in an adult ICU population. **Intensive Care Med,** v. 36, n. 3, p. 444-51, Mar 2010. ISSN 0342-4642.

ELSEVIERS, M. M. et al. Renal replacement therapy is an independent risk factor for mortality in critically ill patients with acute kidney injury. **Crit Care,** v. 14, n. 6, p. R221, 2010. ISSN 1364-8535.

PARK, W. Y. et al. The risk factors and outcome of acute kidney injury in the intensive care units. **Korean J Intern Med,** v. 25, n. 2, p. 181-7, Jun 2010. ISSN 1226-3303 (Print) 1226-3303.

CLEC'H, C. et al. Multiple-center evaluation of mortality associated with acute kidney injury in critically ill patients: a competing risks analysis. **Crit Care,** v. 15, n. 3, p. R128, 2011. ISSN 1364-8535.

DARMON, M. et al. Diagnostic performance of fractional excretion of urea in the evaluation of critically ill patients with acute kidney injury: a multicenter cohort study. **Crit Care,** v. 15, n. 4, p. R178, 2011. ISSN 1364-8535.

GARZOTTO, F. et al. RIFLE-based data collection/management system applied to a prospective cohort multicenter Italian study on the epidemiology of acute kidney injury in the intensive care unit. **Blood Purif,** v. 31, n. 1-3, p. 159-71, 2011. ISSN 0253-5068.

MACEDO, E. et al. Oliguria is an early predictor of higher mortality in critically ill patients. **Kidney Int,** v. 80, n. 7, p. 760-7, Oct 2011. ISSN 0085-2538.

MACEDO, E. et al. Defining urine output criterion for acute kidney injury in critically ill patients. **Nephrol Dial Transplant,** v. 26, n. 2, p. 509-15, Feb 2011. ISSN 0931-0509.

MANDELBAUM, T. et al. Outcome of critically ill patients with acute kidney injury using the Acute Kidney Injury Network criteria. **Crit Care Med,** v. 39, n. 12, p. 2659-64, Dec 2011. ISSN 0090-3493.

MEDVE, L. et al. Epidemiology of acute kidney injury in Hungarian intensive care units: a multicenter, prospective, observational study. **BMC Nephrol,** v. 12, p. 43, 2011. ISSN 1471-2369.

OSTERMANN, M.; CHANG, R. W.Impact of different types of organ failure on outcome in intensive care unit patients with acute kidney injury. **J Crit Care,** v. 26, n. 6, p. 635.e1-635.e10, Dec 2011. ISSN 0883-9441.

PICCINNI, P. et al. Prospective multicenter study on epidemiology of acute kidney injury in the ICU: a critical care nephrology Italian collaborative effort (NEFROINT). **Minerva Anestesiol,** v. 77, n. 11, p. 1072-83, Nov 2011. ISSN 0375-9393.

PROWLE, J. R. et al. Oliguria as predictive biomarker of acute kidney injury in critically ill patients. **Crit Care,** v. 15, n. 4, p. R172, 2011. ISSN 1364-8535.

CLARK, E. et al. Timing the initiation of renal replacement therapy for acute kidney injury in Canadian intensive care units: a multicentre observational study. **Can J Anaesth,** v. 59, n. 9, p. 861-70, Sep 2012. ISSN 0832-610x.

HAN, S. S. et al. Additional role of urine output criterion in defining acute kidney injury. **Nephrol Dial Transplant,** v. 27, n. 1, p. 161-5, Jan 2012. ISSN 0931-0509.

MEDVE, L.; GONDOS, T. Epidemiology of postoperative acute kidney injury in Hungarian intensive care units: an exploratory analysis. **Ren Fail,** v. 34, n. 9, p. 1074-8, 2012. ISSN 0886-022x.

ODUTAYO, A. et al. Epidemiology of acute kidney injury in Canadian critical care units: a prospective cohort study. **Can J Anaesth,** v. 59, n. 10, p. 934-42, Oct 2012. ISSN 0832-610x.

SHASHATY, M. G. et al. African American race, obesity, and blood product transfusion are risk factors for acute kidney injury in critically ill trauma patients. **J Crit Care,** v. 27, n. 5, p. 496-504, Oct 2012. ISSN 0883-9441.

SIGURDSSON, M. I. et al. Acute kidney injury in intensive care units according to RIFLE classification: a population-based study. **Acta Anaesthesiol Scand,** v. 56, n. 10, p. 1291-7, Nov 2012. ISSN 0001-5172.

VAARA, S. T. et al. Population-based incidence, mortality and quality of life in critically ill patients treated with renal replacement therapy: a nationwide retrospective cohort study in Finnish intensive care units. **Crit Care,** v. 16, n. 1, p. R13, 2012. ISSN 1364-8535.

WOHLAUER, M. V. et al. Acute kidney injury and posttrauma multiple organ failure: the canary in the coal mine. **J Trauma Acute Care Surg,** v. 72, n. 2, p. 373-8; discussion 379-80, Feb 2012. ISSN 2163-0755.

ALLEGRETTI, A. S. et al. Continuous renal replacement therapy outcomes in acute kidney injury and end-stage renal disease: a cohort study. **Crit Care,** v. 17, n. 3, p. R109, 2013. ISSN 1364-8535.

ALSULTAN, M. A. The renal recovery of critically ill patients with acute renal failure requiring dialysis. **Saudi J Kidney Dis Transpl,** v. 24, n. 6, p. 1175-9, Nov 2013. ISSN 1319-2442 (Print) 1319-2442.

FUCHS, L. et al. Severity of acute kidney injury and two-year outcomes in critically ill patients. **Chest,** v. 144, n. 3, p. 866-75, Sep 2013. ISSN 0012-3692.

LEGRAND, M. et al. Association between systemic hemodynamics and septic acute kidney injury in critically ill patients: a retrospective observational study. **Crit Care,** v. 17, n. 6, p. R278, 2013. ISSN 1364-8535.

NISULA, S. et al. Incidence, risk factors and 90-day mortality of patients with acute kidney injury in Finnish intensive care units: the FINNAKI study. **Intensive Care Med,** v. 39, n. 3, p. 420-8, Mar 2013. ISSN 0342-4642.

POUKKANEN, M. et al. Acute kidney injury in patients with severe sepsis in Finnish Intensive Care Units. **Acta Anaesthesiol Scand,** v. 57, n. 7, p. 863-72, Aug 2013. ISSN 0001-5172.

POUKKANEN, M. et al. Hemodynamic variables and progression of acute kidney injury in critically ill patients with severe sepsis: data from the prospective observational FINNAKI study. **Crit Care,** v. 17, n. 6, p. R295, 2013. ISSN 1364-8535.

DOI, K. et al. Repulsive guidance cue semaphorin 3A in urine predicts the progression of acute kidney injury in adult patients from a mixed intensive care unit. **Nephrol Dial Transplant,** v. 29, n. 1, p. 73-80, Jan 2014. ISSN 0931-0509.

HAN, S. S. et al. Proteinuria and hematuria are associated with acute kidney injury and mortality in critically ill patients: a retrospective observational study. **BMC Nephrol,** v. 15, p. 93, 2014. ISSN 1471-2369.

LINDER, A. et al. Small acute increases in serum creatinine are associated with decreased long-term survival in the critically ill. **Am J Respir Crit Care Med,** v. 189, n. 9, p. 1075-81, May 1 2014. ISSN 1073-449x.

SHINJO, H. et al. Comparison of kidney disease: improving global outcomes and acute kidney injury network criteria for assessing patients in intensive care units. **Clin Exp Nephrol,** v. 18, n. 5, p. 737-45, Oct 2014. ISSN 1342-1751.

UDY, A. A. et al. Augmented renal clearance in the ICU: results of a multicenter observational study of renal function in critically ill patients with normal plasma creatinine concentrations*. **Crit Care Med,** v. 42, n. 3, p. 520-7, Mar 2014. ISSN 0090-3493.

BOUCHARD, J. et al. A Prospective International Multicenter Study of AKI in the Intensive Care Unit. **Clin J Am Soc Nephrol,** v. 10, n. 8, p. 1324-31, Aug 7 2015. ISSN 1555-9041.

HARRIS, D. G. et al. Epidemiology and outcomes of acute kidney injury in critically ill surgical patients. **J Crit Care,** v. 30, n. 1, p. 102-6, Feb 2015. ISSN 0883-9441.

RIMES-STIGARE, C. et al. Evolution of chronic renal impairment and long-term mortality after de novo acute kidney injury in the critically ill; a Swedish multi-centre cohort study. **Crit Care,** v. 19, p. 221, May 6 2015. ISSN 1364-8535.

VANMASSENHOVE, J. et al. Prognostic robustness of serum creatinine based AKI definitions in patients with sepsis: a prospective cohort study. **BMC Nephrology**, London**,** v. 16, p. 112, 07/22 03/15/received 07/06/accepted 2015. ISSN 1471-2369.

MATALOUN, S. E. et al. Incidence, risk factors and prognostic factors of acute renal failure in patients admitted to an intensive care unit^ien. **Braz. j. med. biol. res,** v. 39, n. 10, p. 1339-1347, 10 2006. ISSN 0100-879X.

SILVA JÚNIOR, G. B. D. et al. Risk factors for death among critically ill patients with acute renal failure. **São Paulo med. j,** v. 124, n. 5, p. 257-263, 09 2006. ISSN 1516-3180.

CHOW, Y. W.; LIM, B. B.; HOOI, L. S. Acute renal failure in the same hospital ten years apart. **Med J Malaysia,** v. 62, n. 1, p. 27-32, Mar 2007. ISSN 0300-5283 (Print)0300-5283.

DAHER, E. F. et al. Acute kidney injury in an infectious disease intensive care unit - an assessment of prognostic factors. **Swiss Med Wkly,** v. 138, n. 9-10, p. 128-33, Mar 8 2008. ISSN 1424-7860 (Print)0036-7672.

LIMA, R. S. et al. Comparison between early and delayed acute kidney injury secondary to infectious disease in the intensive care unit. **Int Urol Nephrol,** v. 40, n. 3, p. 731-9, 2008. ISSN 0301-1623 (Print)0301-1623.

FERNANDES, N. M. D. S. et al. Uso do escore prognóstico APACHE II e ATN-ISS em insuficiência renal aguda tratada dentro e fora da unidade de terapia intensiva. **Revista da Associação Médica Brasileira,** v. 55, p. 434-441, 2009. ISSN 0104-4230.

FRIEDERICKSEN, D. V. et al. Acute renal failure in the medical ICU still predictive of high mortality. **S Afr Med J,** v. 99, n. 12, p. 873-5, Dec 2009. ISSN 0256-9574 (Print).

CHANG, C. H. et al. Acute kidney injury classification: comparison of AKIN and RIFLE criteria. **Shock,** v. 33, n. 3, p. 247-52, Mar 2010. ISSN 1073-2322.

MACCARIELLO, E. et al. SAPS 3 scores at the start of renal replacement therapy predict mortality in critically ill patients with acute kidney injury. **Kidney Int,** v. 77, n. 1, p. 51-6, Jan 2010. ISSN 0085-2538.

BALUSHI, F. et al. Acute kidney injury in a teaching hospital in Oman. **Saudi J Kidney Dis Transpl,** v. 22, n. 4, p. 825-8, Jul 2011. ISSN 1319-2442 (Print)1319-2442.

PONCE, D. et al. Injúria renal aguda em unidade de terapia intensiva: estudo prospectivo sobre a incidência, fatores de risco e mortalidade. **Revista Brasileira de Terapia Intensiva,** v. 23, p. 321-326, 2011. ISSN 0103-507X.

FONSECA RUIZ, N. J. et al. Renal injury study in critical ill patients in accordance with the new definition given by the Acute Kidney Injury Network. **J Crit Care,** v. 26, n. 2, p. 206-12, Apr 2011. ISSN 0883-9441.

SAMIMAGHAM, H. R. et al. Acute kidney injury in intensive care unit: incidence, risk factors and mortality rate. **Saudi J Kidney Dis Transpl,** v. 22, n. 3, p. 464-70, May 2011. ISSN 1319-2442 (Print)1319-2442.

ALVES, C. M. P.; BARROS, M. D. C.; FIGUEIREDO, P. V. T. Different approaches in the detection of acute renal dysfunction in serious patients. 2012 2012.

CHEN, T. H. et al. Acute kidney injury biomarkers for patients in a coronary care unit: a prospective cohort study. **PLoS One,** v. 7, n. 2, p. e32328, 2012. ISSN 1932-6203.

DAHER, E. F. et al. Differences in community, hospital and intensive care unit-acquired acute kidney injury: observational study in a nephrology service of a developing country. **Clin Nephrol,** v. 78, n. 6, p. 449-55, Dec 2012. ISSN 0301-0430.

LAI, C. F. et al. Kidney function decline after a non-dialysis-requiring acute kidney injury is associated with higher long-term mortality in critically ill survivors. **Crit Care,** v. 16, n. 4, p. R123, 2012. ISSN 1364-8535.

WAHRHAFTIG, K. D. M. et al. RIFLE Classification: prospective analysis of the association with mortality in critical ill patiants. **J. Bras. Nefrol.,** v. 34, n. 4, p. 369-377, 12/2012 2012. ISSN 0101-2800.

ZHOU, J. et al. Risk factors for the prognosis of acute kidney injury under the Acute Kidney Injury Network definition: a retrospective, multicenter study in critically ill patients. **Nephrology (Carlton),** v. 17, n. 4, p. 330-7, May 2012. ISSN 1320-5358.

DALBONI, M. A. et al. Tumour necrosis factor-alpha plus interleukin-10 low producer phenotype predicts acute kidney injury and death in intensive care unit patients. **Clin Exp Immunol,** v. 173, n. 2, p. 242-9, Aug 2013. ISSN 0009-9104.

LEVI, T. M. et al. Comparison of the RIFLE, AKIN and KDIGO criteria to predict mortality in critically ill patients. **Rev Bras Ter Intensiva,** v. 25, n. 4, p. 290-6, Oct-Dec 2013. ISSN 0103-507x.

COSTA E SILVA, V. T. et al. Nephrology referral and outcomes in critically ill acute kidney injury patients. **PLoS One,** v. 8, n. 8, p. e70482, 2013. ISSN 1932-6203.

SINGH, T. B. et al. Hospital-acquired acute kidney injury in medical, surgical, and intensive care unit: A comparative study. **Indian J Nephrol,** v. 23, n. 1, p. 24-9, Jan 2013. ISSN 0971-4065 (Print)0971-4065.

DAHER, E. D. F. et al. Acute kidney injury in a tropical country: a cohort study of 253 patients in an infectious diseases intensive care unit^ien. **Rev. Soc. Bras. Med. Trop,** v. 47, n. 1, p. 86-89, 01 2014. ISSN 0037-8682.

LUO, X. et al. A comparison of different diagnostic criteria of acute kidney injury in critically ill patients. **Crit Care,** v. 18, n. 4, p. R144, 2014. ISSN 1364-8535.

MORALES-BUENROSTRO, L. E. et al. Hsp72 is a novel biomarker to predict acute kidney injury in critically ill patients. **PLoS One,** v. 9, n. 10, p. e109407, 2014. ISSN 1932-6203.

PENG, Q.; ZHANG, L.; AI, Y. Epidemiology of acute kidney injury in intensive care septic patients based on the KDIGO guidelines. **Chin Med J (Engl),** v. 127, n. 10, p. 1820-6, 2014. ISSN 0366-6999 (Print)0366-6999.

WIJEWICKRAMA, E. S. et al. Incidences and clinical outcomes of acute kidney injury in ICU: a prospective observational study in Sri Lanka. **BMC Res Notes,** v. 7, p. 305, 2014. ISSN 1756-0500.

BENTATA, Y. et al. Acute kidney injury according to KDIGO stages and maternal mortality in the intensive care unit. **Intensive Care Medicine,** v. 41, n. 3, p. 555-556, 2015. ISSN 1432-1238.

BOUCHARD, J. et al. A Prospective International Multicenter Study of AKI in the Intensive Care Unit. **Clin J Am Soc Nephrol,** v. 10, n. 8, p. 1324-31, Aug 7 2015. ISSN 1555-9041.

HEEGARD, K. D. et al. Early acute kidney injury in military casualties. **J Trauma Acute Care Surg,** v. 78, n. 5, p. 988-93, May 2015. ISSN 2163-0755.

MD RALIB, A.; MAT NOR, M. B. Acute kidney injury in a Malaysian intensive care unit: Assessment of incidence, risk factors, and outcome. **J Crit Care,** v. 30, n. 3, p. 636-42, Jun 2015a. ISSN 0883-9441.

SANTOS, P. R.; MONTEIRO, D. L. Acute kidney injury in an intensive care unit of a general hospital with emergency room specializing in trauma: an observational prospective study. **BMC Nephrol,** v. 16, p. 30, Mar 19 2015. ISSN 1471-2369.
